# Guideline-Concordant Answer Quality of Five Large Language Models on Indication Criteria for Total Hip Arthroplasty in Hip Osteoarthritis: A Single-Run Benchmark Against the 2021 German S3 Guideline (EKIT-Hip)

**DOI:** 10.3390/jcm15207944

**Published:** 2026-10-14

**Authors:** Obada Alalman, Max J. Scheyerer, Wajjahat Syed, Maher Ghandour, Matthias Hermasch, Christoph Beyersdorf, Mohamad Agha Mahmoud, Carl Neuerburg, Koroush Kabir, Ümit Mert

**Affiliations:** 1 Department of Orthopedic and Trauma Surgery, Helios University Hospital, University Witten/Herdecke, 42283 Wuppertal, Germany; obada.alman.97@gmail.com (O.A.); wajjahat.syed@uni-wh.de (W.S.); mghandourmd@gmail.com (M.G.); m.agha.mahmoud@gmail.com (M.A.M.); koroush.kabir@helios-gesundheit.de (K.K.); 2 Department of Orthopedics and Trauma Surgery, Medical Faculty, University Duesseldorf, 40225 Duesseldorf, Germany; max.scheyerer@med.uni-duesseldorf.de (M.J.S.); matthiasandreas.hermasch@med.uni-duesseldorf.de (M.H.); christoph.beyersdorf@med.uni-duesseldorf.de (C.B.); carl.neuerburg@med.uni-duesseldorf.de (C.N.)

**Keywords:** Osteoarthritis, hip, large language models, ChatGPT, GPT-4o, Gemini, artificial intelligence, benchmarking, clinical practice guidelines, total hip arthroplasty, DGOU

## Abstract

**Background/Objectives**: Hip osteoarthritis (OA) is a highly prevalent musculoskeletal disorder associated with substantial functional impairment and healthcare burden. Although evidence-based clinical practice guidelines exist, including the 2021 guideline of the German Society for Orthopaedics and Trauma Surgery (DGOU), their implementation in routine practice remains incomplete. Large language models (LLMs) are increasingly consulted for guideline-related information, but the concordance of their answers with guideline recommendations is uncertain. This benchmark study evaluated the guideline concordance, comprehensiveness and length of answers generated by five widely used LLMs to questions derived from the 2021 German S3 guideline on evidence- and consensus-based indication criteria for total hip arthroplasty in hip OA (EKIT-Hip; AWMF register no. 187-001; lead society DGOU). It did not evaluate the effectiveness or safety of clinical decision support. **Methods**: In a cross-sectional, single-run benchmark study, 26 structured, context-free questions covering five of the six thematic complexes of the guideline (diagnosis and imaging; assessment of the level of suffering; evaluation of alternative therapeutic options; contraindications; optimisation of modifiable risk factors) were derived from the guideline recommendations and submitted between 9 November 2024 and 25 February 2025 through the consumer web interfaces of five LLM-based systems (GPT-4, GPT-4o, GPT-4o mini, Gemini Advanced and Consensus Premium), yielding 130 responses (one per system–question pair, each in a newly initiated chat session). Responses were evaluated by three board-certified orthopaedic surgeons blinded to model identity, using a four-point accuracy scale and a five-point comprehensiveness scale. Only a single consensus score per response was recorded, so inter-rater reliability could not be assessed. Response length was quantified in words and characters and benchmarked against the corresponding guideline text. Because all systems answered the same questions, ratings were analysed as paired, question-clustered data with cumulative-link mixed models (random intercept for question), Friedman and Cochran’s Q tests and question-level cluster-bootstrap confidence intervals; unpaired analyses were retained as sensitivity analyses. **Results**: Accuracy differed across systems (cumulative-link mixed model, likelihood-ratio χ^2^(4) = 49.0, *p* < 0.0001; paired Friedman χ^2^(4) = 41.7, *p* < 0.0001; unpaired Kruskal–Wallis sensitivity analysis H = 35.6, *p* < 0.0001). Mean accuracy was highest for GPT-4o (3.69 ± 0.55) and GPT-4 (3.62 ± 0.57), followed by GPT-4o mini (3.27 ± 0.78), Gemini Advanced (3.08 ± 0.84) and Consensus Premium (2.54 ± 0.71). Responses rated good or excellent accounted for 25/26 (96.2%) of GPT-4o and GPT-4 responses versus 15/26 (57.7%) for Consensus Premium. Comprehensiveness followed the same ordering (H = 33.0, *p* < 0.0001, ε^2^ = 0.23), with GPT-4o achieving the highest mean score (4.31 ± 0.79). In the cumulative-link mixed model, the odds of a higher accuracy rating relative to Consensus Premium were 51.8 (95% CI 12.4–215.9) for GPT-4o and 35.4 (95% CI 9.2–136.4) for GPT-4; GPT-4o and GPT-4 did not differ (OR 1.47, multiplicity-adjusted 95% CI 0.23–9.21), and their paired mean difference in accuracy (0.08 points, 90% CI −0.11 to 0.26) lay within an equivalence margin of ±0.5 points. Responses rated poor were observed for Consensus Premium (2/26) and Gemini Advanced (1/26) and not for the ChatGPT variants (0/26 each; exact 95% CI 0–13.2%), so a clinically relevant error rate cannot be excluded for any system. Response length differed markedly (Friedman χ^2^ = 47.3, *p* < 0.0001), ranging from 32.4 ± 11.5 words for Consensus Premium to 77.8 ± 24.9 words for GPT-4o, corresponding to 1.29-fold and 3.19-fold the length of the source guideline text, respectively. **Conclusions**: GPT-4o and GPT-4 received the highest ratings for accuracy and comprehensiveness in this single-run benchmark of static, context-free questions. These data describe guideline-concordant answer quality only; they do not establish clinical decision-support effectiveness or safety and do not support unsupervised clinical use of any evaluated system. Evaluation with standardised patient cases, repeated version-controlled runs, clinician-in-the-loop designs and prospective safety endpoints is required.

## 1. Introduction

Osteoarthritis (OA) is a leading cause of musculoskeletal pain and disability, particularly affecting the knee and hip joints, and imposes a considerable societal burden through work absenteeism, early retirement and related healthcare costs [1]. Hip OA (coxarthrosis) is highly prevalent among older adults and is one of the most common musculoskeletal disorders encountered in orthopaedic and trauma departments [2,3]. OA is generally classified as primary or secondary: primary OA is idiopathic, typically occurs in older adults and often involves multiple joints, whereas secondary OA usually affects a single joint and arises from an identifiable cause such as trauma or another joint disorder [4].

Multiple clinical practice guidelines exist to support clinicians in managing OA; however, despite broad agreement across professional societies, their dissemination and implementation remain limited, resulting in suboptimal uptake in clinical practice [5]. Guideline-recommended care is delivered to fewer than 40% of patients [6], and non-pharmacological conservative treatments may be underused in patients with hip and knee OA referred for orthopaedic consultation [7]. Moreover, most evidence-based guidelines offer limited guidance on implementation and lack consensus on appropriate management, contributing to low adherence in clinical practice [8]. Against this background, the German Society for Orthopaedics and Trauma Surgery (Deutsche Gesellschaft für Orthopädie und Unfallchirurgie, DGOU) coordinated an S3 guideline, published in 2021 under the auspices of the Association of the Scientific Medical Societies in Germany (AWMF), that defines evidence- and consensus-based indication criteria for total hip arthroplasty in hip OA (EKIT-Hip; AWMF register no. 187-001) [9,10]. Its 31 recommendations are organised into six thematic complexes that follow the sequence of the indication decision: confirmation of the diagnosis, the individual burden of disease, alternative treatment options, contraindications, modifiable risk factors and shared decision-making [11]. The recommendations are based on a systematic review of the evidence and structured expert consensus; nevertheless, successful implementation of evidence-based guidelines remains challenging.

Educational approaches such as guideline dissemination and continuing medical education primarily promote awareness and acceptance, but the process of adoption into clinical practice remains poorly understood [12]. While integrating support systems into busy clinical settings may initially disrupt workflow, such systems have the potential to improve efficiency and patient outcomes by promoting adherence to evidence-based practice [12]. Artificial intelligence (AI) is one such support system, increasingly used to assist healthcare professionals in both clinical practice and research [13].

AI in medical practice continues to evolve. By learning complex patterns from large datasets using layered algorithms, AI systems improve their performance through repeated training [14]. The application of AI in healthcare, including orthopaedics, is expanding, with research highlighting its potential to support clinical decision-making, risk assessment and surgical planning [15]. Given the growing impact of AI and machine learning on society, their integration into the education of domain experts is essential, particularly in medicine, where incorrect decisions can have fatal consequences [16]. AI demonstrates high diagnostic accuracy and rapid processing in fields such as radiology, pathology and dermatology, as well as in surgical specialties such as spinal surgery; consequently, when combined with clinician expertise, it can further enhance diagnostic performance by improving accuracy, increasing efficiency and enabling personalised decision-making [14,17].

Rapid advances in AI have made large language models (LLMs) a central topic of investigation. LLMs are deep learning models trained on extensive text corpora to capture the patterns and rules of language, enabling them to understand and generate text with high accuracy across diverse applications [18]. LLM-based agents have emerged as transformative tools in biomedicine, evolving from simple utilities into powerful enablers of innovation with applications in clinical decision support, medical education and research, while reshaping research, accelerating discovery and improving clinical workflows, although challenges related to hallucination, interpretability, privacy and responsible deployment remain [19]. Well-known models include OpenAI’s ChatGPT (GPT-4, GPT-4o, GPT-4o mini), Google’s Gemini and the Consensus platform, all of which have been investigated in previous healthcare studies [20].

Collectively, there is an increasing need for innovative tools to support the translation of evidence into clinical practice, with implementation requiring careful validation and oversight. AI has the potential to assist clinicians in diagnosing hip OA, predicting disease progression and facilitating adherence to evidence-based management, particularly in complex or high-volume clinical settings [21]. Whether LLMs can support guideline-based care in practice, however, depends first on whether their answers reproduce guideline content correctly. This is a necessary but not sufficient condition that must be established before clinical decision support, workflow integration or patient outcomes can be evaluated, and it requires systematic, specialty-specific evaluation.

Evidence from other domains of digital health information shows why such evaluation must be independent and multidimensional. In a cross-sectional study of 200 weight-management short videos on TikTok and Bilibili, Du et al. rated quality and reliability independently with the Global Quality Score and the DISCERN instrument and combined an XGBoost classifier with SHAP explanations to build an explainable quality-identification tool [22]. Reliability was low on both platforms, engagement metrics such as likes were only weakly associated with quality, and duration contributed to the prediction of quality only in a non-linear, threshold-dependent way [22]. By analogy, the fluency, length or apparent authority of an LLM answer cannot be taken as evidence of its scientific accuracy; guideline concordance, completeness and potential for harm must be rated separately against an explicit reference standard.

To our knowledge, the concordance of contemporary LLM answers with the German S3 indication criteria for total hip arthroplasty has not been benchmarked. We therefore conducted a cross-sectional, single-run benchmark of five widely used LLM-based systems using 26 structured questions covering five of the six thematic complexes of the EKIT-Hip guideline. The study was designed to measure guideline-concordant answer quality, not the effectiveness or safety of LLM-based clinical decision support. We hypothesised that (i) guideline concordance and comprehensiveness would differ significantly between models, and (ii) response length would be associated with, but not by itself explain, rated answer quality (exploratory).

## 2. Materials and Methods

### 2.1. Study Design and Setting

A cross-sectional, single-run benchmark study was conducted between 9 November 2024 and 25 February 2025 at the Department of Trauma Surgery, Orthopedics and Sports Medicine, Helios University Clinic Wuppertal, University of Witten/Herdecke, Germany. The primary objective was to assess the accuracy and comprehensiveness of clinical recommendations generated by five LLMs, benchmarked against the recommendations of the 2021 S3 guideline “Evidence- and consensus-based indication criteria for total hip arthroplasty in hip osteoarthritis” (EKIT-Hip; hereafter “the guideline”) [9,10]. Response length was assessed as a secondary, descriptive outcome. The study is reported in accordance with the principles of the STROBE statement for cross-sectional studies. Because the unit of analysis is a model-generated answer to a context-free question rather than a patient or a clinical decision, the study measures guideline-concordant answer quality; it was not designed to assess clinical decision-support effectiveness, clinician–model interaction, workflow, patient outcomes or clinical safety. An overview of the study workflow is provided in Figure 1.

### 2.2. Ethical Considerations

This study did not involve human participants, human tissue or identifiable patient data, and therefore did not require approval by an institutional review board or written informed consent. All LLMs evaluated were publicly accessible and were used in accordance with their standard terms of service. The study adhered to all relevant ethical principles governing research in healthcare settings and to the Declaration of Helsinki where applicable.

### 2.3. Guideline Source and Development of the Question Set

A total of 26 structured clinical questions were derived from the 2021 S3 guideline “Evidenz- und konsensbasierte Indikationskriterien zur Hüfttotalendoprothese bei Coxarthrose (EKIT-Hüfte)” (AWMF register no. 187-001, version 1.0, published 12 April 2021; next scheduled review 31 December 2028; lead society DGOU) [9,10]. The formal scope of this guideline is the definition of evidence- and consensus-based indication criteria for total hip arthroplasty in hip OA; it is not a general guideline on the diagnosis and treatment of hip OA. Its 31 recommendations are organised into six thematic complexes [11]. Questions were constructed for the first five complexes, which were adopted as the five analysis domains:

- Diagnosis and imaging (guideline recommendations 4.1.1–4.1.8; 8 questions).
- Assessment of the patient’s level of suffering (recommendations 4.2.1–4.2.4; 4 questions).
- Evaluation of alternative therapeutic options (recommendations 4.3.1–4.3.3; 3 questions).
- Contraindications (recommendations 4.4.1–4.4.4; 3 questions).
- Optimisation of modifiable risk factors (recommendations 4.5.1–4.5.8; 8 questions).

The sixth thematic complex, shared (participatory) decision-making (Partizipative Entscheidungsfindung), was not operationalised because its recommendations concern the process of patient–clinician communication and preference elicitation rather than content that can be checked against a single guideline statement in a context-free question. The question set therefore does not cover the full guideline, and the results do not apply to shared decision-making. Each question was mapped to one numbered guideline recommendation, whose text served as the reference answer; the recommendation number for every question is listed in Supplementary Tables S1 and S5 gives the full German text of each recommendation with its grade of recommendation, level of evidence and consensus strength, together with an English translation. Questions were phrased as open clinical questions in natural language, without prompting the model to cite the guideline and without patient-specific context; they therefore test the reproduction of guideline content rather than case-based clinical reasoning. Questions and reference answers were formulated by the investigators, and the distribution of items across domains is summarised in Table 1.

### 2.4. Large Language Models and Query Procedure

Each question was independently submitted to five LLM platforms: GPT-4, GPT-4o and GPT-4o mini (OpenAI, San Francisco, CA, USA), Gemini Advanced (Gemini 1.5 Pro; Google LLC, Mountain View, CA, USA) and the Consensus platform (Consensus Premium; Consensus AI, Boston, MA, USA). ChatGPT models were accessed through the OpenAI web platform (chatgpt.com) under a paid subscription; Gemini Advanced was accessed through the Google AI web interface; and Consensus Premium was accessed through its web platform under the Premium plan. All queries were entered through the respective web application interfaces with default settings and without user-activated plugins or third-party tools. Because these were consumer products rather than fixed model versions, the comparison is between product configurations: Consensus Premium generates literature-grounded answers from retrieved research papers with citations, Gemini Advanced may draw on built-in web retrieval, and the ChatGPT interfaces apply their own undisclosed system prompts and sampling parameters. Model effects are therefore confounded with product-specific retrieval, citation and interface policies. System prompts, temperature and other sampling parameters could neither be set nor observed in the web interfaces, and the interfaces displayed only the product and model labels reported above, without dated snapshot identifiers.

To reduce carry-over effects from preceding interactions, every query was submitted within a newly initiated chat session, and no follow-up prompts, clarifications or regenerations were permitted; the first output returned by the model was retained. A new chat session reduces context carry-over but does not address the stochastic variation between repeated generations of the same prompt; the primary benchmark therefore describes a single sample from each system’s output distribution. This yielded 130 responses in total (26 questions × 5 models). All responses were recorded verbatim, stripped of any model-identifying formatting, assigned a random alphanumeric identifier and securely archived before evaluation. Microsoft Word and Microsoft Excel (Microsoft Corporation, Redmond, WA, USA) were used to organise, structure and anonymise the data for expert evaluation. Data collection extended over approximately 15 weeks, during which providers may have updated the underlying models without notice. Version drift within the collection period can therefore not be excluded. Several of the evaluated versions have since been retired or replaced in the consumer interfaces, so an exact re-run of the original configurations is no longer possible.

### 2.5. Outcome Measures and Expert Evaluation

All model responses were evaluated by a panel of three board-certified orthopaedic surgeons with subspecialty expertise in hip and joint disorders and heterogeneous clinical experience. All reviewers were blinded to model identity and to the responses’ order of generation. Reviewers assessed each response for concordance with the 2021 DGOU guideline and assigned scores using predefined ordinal scales; discrepancies were resolved by structured discussion until agreement was reached, and a single consensus score per response was recorded for analysis. Because the assessors’ individual ratings were not recorded separately, inter-rater reliability could not be quantified, and the success of blinding was not assessed (Section 4.1).

#### 2.5.1. Accuracy Rating Scale

Accuracy was the primary outcome and was rated on a four-point ordinal scale adapted from previously validated frameworks [20]:

- Poor (1): contains major factual errors that could mislead clinicians or potentially lead to harmful decisions.
- Moderate (2): includes moderate inaccuracies; unlikely to cause harm but requires clarification for safe clinical use.
- Good (3): contains only minor inaccuracies; generally safe and clinically applicable with minimal clarification.
- Excellent (4): fully accurate and consistent with guideline-based recommendations, requiring no clarification.

For clinically oriented reporting, responses rated good or excellent (≥3) were pre-specified as “guideline-concordant”, and responses rated poor or moderate (≤2) as “sub-optimal”.

#### 2.5.2. Comprehensiveness Rating Scale

Comprehensiveness was a secondary outcome and was rated on a five-point ordinal scale [20]:

- Not comprehensive (1): lacks essential clinical information and is substantially inadequate.
- Slightly comprehensive (2): provides only minimal, basic details.
- Moderately comprehensive (3): offers an acceptable level of detail but omits certain important nuances.
- Comprehensive (4): covers most key elements recommended in the guideline.
- Very comprehensive (5): provides extensive, nuanced information closely aligned with guideline recommendations.

Responses rated comprehensive or very comprehensive (≥4) were pre-specified as “highly comprehensive”.

Both scales were adapted from a previous guideline-benchmarking study [20], with the anchors defined above for each level. Because assessors saw the full answer when rating comprehensiveness, these ratings may be susceptible to a length halo effect; the relationship between length and ratings was therefore analysed only as an exploratory, question-level analysis (Section 2.6).

#### 2.5.3. Response Length Measurement

Response length was assessed as a descriptive outcome. For each of the 130 responses, the total word count and total character count were extracted programmatically. To place verbosity in clinical context, each response was benchmarked against the word and character count of the corresponding DGOU guideline recommendation, and a per-question verbosity ratio (model length divided by guideline length) was computed. Because guideline recommendations are deliberately concise, this ratio describes elaboration relative to the guideline wording and is not a measure of clinical usability.

### 2.6. Statistical Analysis

Descriptive data are reported as mean ± standard deviation and median with interquartile range (IQR), and categorical data as counts and percentages. Because every system answered the same 26 questions, the 130 ratings form a paired, question-clustered design, and all primary inferential analyses account for this structure. The primary analysis for accuracy and comprehensiveness was a cumulative-link (proportional-odds) mixed model with system as a fixed effect (Consensus Premium as reference) and a random intercept for question; models additionally including domain as a fixed effect, or a random intercept for domain, were fitted as sensitivity analyses. The proportional-odds assumption was assessed by comparing each model with a partial proportional-odds (nominal-effects) alternative using a likelihood-ratio test. Odds ratios are reported with Wald 95% confidence intervals (CI) and with percentile CIs from a question-level cluster bootstrap (2000 resamples of the 26 questions, keeping all responses to a resampled question together). Pairwise contrasts are reported as effect estimates with Bonferroni-adjusted 95% CIs and Holm-adjusted *p*-values rather than *p*-values alone. Differences that were not statistically significant were not interpreted as equivalence; for GPT-4 versus GPT-4o, the paired difference in mean ratings was assessed with two one-sided tests (TOST, 90% CI) against an equivalence margin of ±0.5 scale points (half of one rating category), defined for this revision.

As a distribution-free paired sensitivity analysis, accuracy and comprehensiveness were compared with the Friedman test (Kendall’s W as effect size) followed by Conover post hoc tests with Holm correction. Dichotomised outcomes (good or excellent accuracy; comprehensive or very comprehensive; major error) were analysed with Cochran’s Q test, with pairwise exact McNemar tests (Holm-adjusted) and paired differences in proportions with question-level cluster-bootstrap 95% CIs, and with mixed-effects logistic regression (random intercept for question) where events were sufficiently frequent. Model-specific proportions of major errors are reported with exact Clopper–Pearson 95% CIs; where no events were observed, the upper confidence bound indicates the largest error rate compatible with the data. The stability of the ranking of systems by mean score was assessed with a question-level bootstrap (10,000 resamples), recording how often each system ranked first and how often pairs of systems kept their order. Response-length data were analysed with the Friedman test and Conover post hoc tests with Holm correction, and each system’s response length was compared with the corresponding guideline text using the Wilcoxon signed-rank test. The analyses of the original submission, which treated the 130 responses as independent (Kruskal–Wallis tests with Dunn post hoc comparisons and Cliff’s delta, chi-square tests with Wilson CIs, and a proportional-odds model without random effects), are retained and labelled as unpaired sensitivity analyses (Section 3.2, Section 3.3 and Section 3.4).

The association between response length and rated quality was examined as an exploratory, question-level analysis using cumulative-link mixed models with log-transformed word count (centred within question) and domain as fixed effects and a random intercept for question, without and with additional adjustment for system, and is displayed in question-level scatterplots with 95% confidence bands. No ordering of the systems by capability was defined before outcome inspection, and the size, training data and inference resources of these proprietary systems are not disclosed; therefore, no trend test across capability tiers was performed, and the model-level rank correlation and the composite performance index of the original submission were removed.

All tests were two-sided and statistical significance was defined as *p* < 0.05. Analyses were performed in Python 3.11 (Python Software Foundation, Wilmington, DE, USA) using SciPy, statsmodels and scikit-posthocs and, for the mixed-effects and bootstrap analyses, in R 4.3.3 (R Foundation for Statistical Computing, Vienna, Austria) with the packages ordinal (2023.12-4) and lme4 (1.1-35) (analysis code in Supplementary Code S1), with confirmatory analyses in GraphPad Prism version 8.0.1 (GraphPad Software, San Diego, CA, USA). Figures were generated with Matplotlib. Significance is annotated on the figures as follows: * *p* < 0.05; ** *p* < 0.01; *** *p* < 0.001; **** *p* < 0.0001.

## 3. Results

### 3.1. Response Length

The number of words per response differed substantially between the evaluated LLMs (Figure 2A, Table 2). GPT-4o generated the longest responses (77.8 ± 24.9 words; median 78.0, IQR 62.8–98.8), followed by GPT-4 (62.1 ± 24.3 words) and GPT-4o mini (57.5 ± 17.2 words). Gemini Advanced produced shorter answers (40.0 ± 13.7 words), while Consensus Premium generated the most concise responses overall (32.4 ± 11.5 words). The Friedman test confirmed a highly significant difference across models (χ^2^(4) = 47.3, *p* < 0.0001, Kendall’s W = 0.45). Conover post hoc testing showed that all three ChatGPT variants produced significantly longer responses than Consensus Premium (all *p* < 0.0001) and Gemini Advanced (*p* ≤ 0.003), and that GPT-4o was significantly more verbose than GPT-4 (*p* = 0.039); GPT-4 and GPT-4o mini did not differ (*p* = 0.816).

Character counts mirrored these findings (Figure 2B). GPT-4o produced the longest outputs (512.1 ± 147.3 characters), followed by GPT-4 (400.3 ± 141.4) and GPT-4o mini (384.5 ± 115.9), with Gemini Advanced (271.5 ± 99.4) and Consensus Premium (217.2 ± 80.4) producing the shortest outputs (Friedman χ^2^(4) = 46.4, *p* < 0.0001, Kendall’s W = 0.45).

Benchmarking against the source guideline placed this variation in context (Figure 2C,D). The corresponding DGOU recommendations averaged 27.8 ± 10.0 words and 165.3 ± 69.1 characters. Consensus Premium responses were closest in length to the guideline text (1.29 ± 0.60 times as long; Wilcoxon *p* = 0.141), whereas Gemini Advanced (1.59 ± 0.80×, *p* = 0.0009), GPT-4o mini (2.28 ± 0.93×, *p* < 0.0001), GPT-4 (2.53 ± 1.41×, *p* < 0.0001) and GPT-4o (3.19 ± 1.57×, *p* < 0.0001) all elaborated significantly beyond the guideline wording. Domain-level analysis showed that this elaboration was not uniform: it was most pronounced for questions on optimisation of modifiable risk factors (D5), where GPT-4o averaged 94.8 ± 20.7 words against 20.1 ± 3.9 words of guideline text (Friedman *p* < 0.0001), and least pronounced for questions on assessment of the level of suffering (D2), where between-model differences were not significant (*p* = 0.442) (Supplementary Table S2).

### 3.2. Accuracy

Accuracy differed markedly between the evaluated LLMs (Figure 3, Table 3). Mean accuracy scores were highest for GPT-4o (3.69 ± 0.55; 95% CI 3.46–3.88) and GPT-4 (3.62 ± 0.57; 95% CI 3.38–3.81), followed by GPT-4o mini (3.27 ± 0.78; 95% CI 2.96–3.54), Gemini Advanced (3.08 ± 0.84; 95% CI 2.73–3.38) and Consensus Premium (2.54 ± 0.71; 95% CI 2.27–2.81). GPT-4o achieved the highest proportion of responses rated excellent (19/26, 73.1%), followed by GPT-4 (17/26, 65.4%), GPT-4o mini (12/26, 46.2%), Gemini Advanced (9/26, 34.6%) and Consensus Premium (1/26, 3.8%).

In the primary cumulative-link mixed model with a random intercept for question, accuracy differed significantly between systems (likelihood-ratio χ^2^(4) = 49.0, *p* < 0.0001; standard deviation of the question intercept 1.31), and the proportional-odds assumption was not rejected (χ^2^(8) = 6.12, *p* = 0.63). All four systems received higher ratings than Consensus Premium (Holm-adjusted *p* = 0.021 for Gemini Advanced, *p* = 0.002 for GPT-4o mini and *p* < 0.0001 for GPT-4 and GPT-4o), and GPT-4 (*p* = 0.015) and GPT-4o (*p* = 0.004) received higher ratings than Gemini Advanced; GPT-4o also outperformed GPT-4o mini (*p* = 0.034). GPT-4o and GPT-4 did not differ (OR 1.47, Bonferroni-adjusted 95% CI 0.23–9.21; *p* = 0.64), nor did GPT-4o mini and Gemini Advanced (*p* = 0.64) or GPT-4o mini and GPT-4 (*p* = 0.094) (odds ratios in Section 3.4 and Supplementary Tables S3 and S4). The paired Friedman test confirmed the global difference (χ^2^(4) = 41.7, *p* < 0.0001, Kendall’s W = 0.40).

In the unpaired sensitivity analysis, the Kruskal–Wallis test likewise demonstrated a significant overall difference in accuracy across the five models (H(4) = 35.6, *p* < 0.0001) with a large effect size (ε^2^ = 0.25). Dunn post hoc testing with Holm correction showed that GPT-4 (*p* < 0.0001), GPT-4o (*p* < 0.0001) and GPT-4o mini (*p* = 0.010) all outperformed Consensus Premium, and that GPT-4o also outperformed Gemini Advanced (*p* = 0.031). No significant difference was observed between GPT-4 and GPT-4o (*p* = 0.828, δ = 0.07, negligible), a non-significant result that does not establish equivalence; the paired mean difference (GPT-4o minus GPT-4) was 0.08 points (95% CI −0.15 to 0.30; 90% CI −0.11 to 0.26), which lies within the equivalence margin of ±0.5 points (TOST *p* < 0.001). Pairwise effect sizes against Consensus Premium were large for GPT-4o (δ = 0.77), GPT-4 (δ = 0.73) and GPT-4o mini (δ = 0.48) (Figure 3C). Results were unchanged when Bonferroni rather than Holm correction was applied.

Responses rated poor (major factual errors) were observed for Consensus Premium (2/26, 7.7%; exact 95% CI 0.9–25.1%) and Gemini Advanced (1/26, 3.8%; 95% CI 0.1–19.6%); none were observed for the three ChatGPT variants in this single-run sample (0/26 each; 95% CI 0–13.2%). The intervals overlap widely; the data therefore do not show that the ChatGPT variants have a lower true major-error rate, and zero observed events do not imply zero risk. Guideline-concordant responses (rated good or excellent) accounted for 25/26 (96.2%) of GPT-4o and GPT-4 responses, 21/26 (80.8%) of GPT-4o mini responses, 20/26 (76.9%) of Gemini Advanced responses and 15/26 (57.7%) of Consensus Premium responses (Cochran’s Q = 22.2, df = 4, *p* = 0.0002; unpaired χ^2^(4) = 17.6, *p* = 0.0015); in pairwise exact McNemar tests, only GPT-4 and GPT-4o differed from Consensus Premium (38.5 percentage points each, cluster-bootstrap 95% CI 19.2–57.7; Holm-adjusted *p* = 0.020). The proportion of major errors did not differ significantly between systems (Cochran’s Q = 5.33, df = 4, *p* = 0.25). Mixed-effects logistic regression was not informative because of near-complete separation (25/26 responses rated good or excellent for GPT-4 and GPT-4o).

### 3.3. Comprehensiveness

Comprehensiveness followed the same ordering as accuracy (Figure 4, Table 4). GPT-4o achieved the highest mean comprehensiveness score (4.31 ± 0.79; 95% CI 4.00–4.58), followed by GPT-4 (4.08 ± 0.74; 95% CI 3.81–4.35), GPT-4o mini (3.88 ± 0.86), Gemini Advanced (3.38 ± 0.94) and Consensus Premium (3.04 ± 0.87). Responses rated very comprehensive were most frequent for GPT-4o (12/26, 46.2%) and GPT-4 (7/26, 26.9%), and were never observed for Consensus Premium (0/26).

In the primary mixed model, comprehensiveness differed significantly between systems (likelihood-ratio χ^2^(4) = 44.3, *p* < 0.0001; proportional-odds test χ^2^(12) = 5.95, *p* = 0.92). GPT-4, GPT-4o mini and GPT-4o received higher ratings than Consensus Premium (Holm-adjusted *p* < 0.0001, *p* = 0.001 and *p* < 0.0001), and GPT-4 (*p* = 0.008) and GPT-4o (*p* = 0.0001) higher ratings than Gemini Advanced; Gemini Advanced did not differ from Consensus Premium (*p* = 0.27), and GPT-4o did not differ from GPT-4 (OR 2.20, Bonferroni-adjusted 95% CI 0.46–10.44; *p* = 0.31). The paired Friedman test gave χ^2^(4) = 35.6, *p* < 0.0001, Kendall’s W = 0.34. The paired mean difference between GPT-4o and GPT-4 was 0.23 points (95% CI −0.08 to 0.54; 90% CI −0.03 to 0.49; TOST *p* = 0.042 for the ±0.5 margin). The unpaired Kruskal–Wallis sensitivity analysis indicated a significant global difference (H(4) = 33.0, *p* < 0.0001, ε^2^ = 0.23). Dunn post hoc analysis showed that GPT-4o (*p* < 0.0001), GPT-4 (*p* = 0.0006) and GPT-4o mini (*p* = 0.009) produced significantly more comprehensive responses than Consensus Premium, and that GPT-4o (*p* = 0.002) and GPT-4 (*p* = 0.043) also outperformed Gemini Advanced (Table 5). GPT-4 and GPT-4o did not differ significantly (*p* = 0.628, δ = 0.19, small). Effect sizes against Consensus Premium were large for GPT-4o (δ = 0.72), GPT-4 (δ = 0.63) and GPT-4o mini (δ = 0.50). Highly comprehensive responses accounted for 23/26 (88.5%) of GPT-4o and 22/26 (84.6%) of GPT-4 responses, compared with 8/26 (30.8%) for Consensus Premium (Cochran’s Q = 30.0, df = 4, *p* < 0.0001; unpaired χ^2^(4) = 27.5, *p* < 0.0001).

### 3.4. Ordinal Regression Modelling

In the primary cumulative-link mixed model with a random intercept for question, the odds of a higher accuracy rating relative to Consensus Premium were 5.37 for Gemini Advanced (Wald 95% CI 1.70–16.91; cluster-bootstrap 95% CI 1.76–19.83), 9.45 for GPT-4o mini (2.87–31.12; 3.16–47.24), 35.36 for GPT-4 (9.17–136.39; 16.53–137.30) and 51.80 for GPT-4o (12.43–215.88; 17.85–338.80) (question intercept variance 1.73). For comprehensiveness, the corresponding odds ratios were 2.51 (0.87–7.24; 0.91–8.18), 8.77 (2.79–27.57; 2.73–45.02), 15.41 (4.77–49.72; 6.94–56.11) and 33.92 (9.68–118.86; 11.61–166.51) (question intercept variance 1.37). These question-conditional odds ratios are larger than the population-averaged estimates of the unpaired model, as expected once between-question variation is accounted for, and their wide intervals reflect the 26 questions. Adding domain as a fixed or random effect left the estimates virtually unchanged (for example, GPT-4o: accuracy OR 51.1 and 51.4; comprehensiveness OR 33.8 and 33.8) (Supplementary Table S3).

In the unpaired sensitivity analysis, a proportional-odds model without random effects gave the following estimates (Figure 5, Table 6). Relative to Consensus Premium, the odds of receiving a higher accuracy rating were 21.3-fold for GPT-4o (95% CI 6.5–70.1; *p* < 0.0001), 15.2-fold for GPT-4 (95% CI 4.9–47.4; *p* < 0.0001), 5.8-fold for GPT-4o mini (95% CI 2.0–17.0; *p* = 0.001) and 3.6-fold for Gemini Advanced (95% CI 1.3–10.3; *p* = 0.016) (likelihood-ratio χ^2^(4) = 37.9, *p* < 0.0001; McFadden’s pseudo-R^2^ = 0.13). For comprehensiveness, the corresponding odds ratios were 19.4 for GPT-4o (95% CI 6.2–60.3; *p* < 0.0001), 9.6 for GPT-4 (95% CI 3.3–28.1; *p* < 0.0001), 6.1 for GPT-4o mini (95% CI 2.1–17.6; *p* = 0.0008) and 2.0 for Gemini Advanced (95% CI 0.7–5.3; *p* = 0.184) (likelihood-ratio χ^2^(4) = 36.7, *p* < 0.0001; pseudo-R^2^ = 0.11).

### 3.5. Response Length and Rated Quality (Exploratory)

Accuracy, comprehensiveness and response length are displayed together in Figure 6. At the question level, longer responses received higher ratings: per doubling of word count (centred within question, adjusted for domain), the odds of a higher rating were 16.2 (95% CI 6.8–38.7) for accuracy and 33.5 (95% CI 13.2–85.0) for comprehensiveness, and 10.3 (95% CI 3.6–29.7) and 26.9 (95% CI 9.1–79.1) after additional adjustment for system. Question-level scatterplots with 95% confidence bands are shown in Figure 6A,B. These analyses are exploratory and cannot show that longer answers cause higher ratings; because assessors saw the full answer, a length halo effect on comprehensiveness ratings cannot be excluded. GPT-4 received accuracy ratings similar to those of GPT-4o (unpaired δ = 0.07) while producing about 20% fewer words (62.1 vs. 77.8 words), indicating that length alone does not determine rated guideline concordance.

## 4. Discussion

AI has undergone rapid development in recent years, with LLMs demonstrating an increasing ability to process, synthesise and generate complex information across a wide range of domains, particularly in medicine [17,23]. By assessing AI performance on USMLE-style questions, Brin et al. showed that advanced LLMs, particularly GPT-4, achieve superior performance in domains such as communication, ethics and professionalism, suggesting their potential applicability in clinically complex scenarios [24].

Against this background, rigorous validation of the accuracy, completeness and safety of these models in clinical settings becomes increasingly critical as they continue to evolve. This need is underscored by the recent launch of ChatGPT Health on 7 January 2026, which enables integration with medical records and personal health data and signals a rapid transition from research evaluation to real-world deployment [25]. Furthermore, Faiyazuddin et al. showed that AI is rapidly evolving in healthcare, as reflected by the substantial increase in AI-related publications over time, while highlighting its growing role in improving diagnostic accuracy, enabling personalised treatment strategies and enhancing patient outcomes [26].

In this single-run benchmark, we evaluated how closely the answers of five LLM-based systems to 26 context-free questions reproduced the recommendations of the 2021 EKIT-Hip guideline. GPT-4o and GPT-4 received the highest accuracy and comprehensiveness ratings, GPT-4o mini intermediate ratings, and Gemini Advanced and Consensus Premium the lowest, with large differences relative to Consensus Premium (mixed-model odds ratios for a higher accuracy rating 35.4 for GPT-4 and 51.8 for GPT-4o). Gemini Advanced and Consensus Premium more often omitted or simplified core recommendations. Because the size, training data and inference resources of these proprietary systems are not disclosed, and because the comparators differ in product-level retrieval and citation functions, these differences cannot be attributed to model scale or training breadth, and we do not interpret them as a monotonic capability gradient. GPT-4 and GPT-4o did not differ, and their paired difference in mean accuracy lay within ±0.5 rating points.

The distribution of the lowest ratings deserves attention but must be interpreted cautiously. Responses rated poor were observed for Consensus Premium (2/26) and Gemini Advanced (1/26) and not for the ChatGPT variants (0/26 each). With one response per question and 26 questions per system, however, the 95% CI for a zero observed rate extends to 13.2%, and the intervals of all five systems overlap. Our data therefore show only that no such events were observed for the ChatGPT variants in this sample; they do not show that these systems are safer, and they cannot replace clinical safety validation. Likewise, the higher proportion of sub-optimal responses for Consensus Premium (11/26, 42.3%) than for GPT-4o and GPT-4 (1/26 each) is a descriptive finding from a single run whose stability across repeated generations remains to be established.

These findings carry several implications for the potential integration of LLMs into orthopaedic care. Persistent gaps in guideline adherence for hip OA are well documented, particularly in the early use of multimodal conservative therapy [6,7]. Whether systems such as GPT-4o and GPT-4 can support clinicians at the point of care, for example in first-line treatment selection or patient counselling, cannot be inferred from the present data, which contain no patient context, no clinician–model interaction and no clinical endpoints. Importantly, the variation in performance between models underscores that not all systems are equally suitable for clinical consultation, and that any future clinical evaluation must be specific to the system and version tested. This is supported by our findings, in which Gemini Advanced and particularly Consensus Premium showed lower accuracy and completeness than GPT-4-based models. It is partially consistent with Milicevic et al., who likewise demonstrated marked inter-model variability in guideline interpretation, with GPT-4o performing best while Consensus Premium outperformed GPT-4o mini in the context of vertebral osteomyelitis guidelines [20]. Taken together, these observations suggest that LLM performance is not only model-dependent but also task- and domain-dependent, raising important questions regarding how such systems should be validated, selected and safely implemented.

Our results align with and extend existing evaluations of LLMs in medicine. The emergence of foundation models offers new opportunities for medical AI, with strong performance in answering medical questions emerging as a key capability despite the complexity of clinical domains [20,23]. However, Wang et al., in a network meta-analysis of 168 studies covering nearly 36,000 clinical questions and more than 3000 clinical cases, demonstrated that LLM performance varies by clinical task, with GPT-4o showing superior accuracy for objective clinical questions, GPT-4 excelling in open-ended reasoning and human experts remaining superior in top diagnostic accuracy [27]. Our finding that GPT-4o and GPT-4 were statistically indistinguishable on guideline-concordance while differing in verbosity is consistent with this task-dependent picture.

As highlighted by Lee et al., advanced LLMs such as GPT-4 may serve as practical clinical support tools by rapidly synthesising and structuring complex medical information [28]. Consistent with this, Milicevic et al. demonstrated that, among four LLMs evaluated for guideline interpretation in vertebral osteomyelitis, GPT-4o achieved the highest accuracy, completeness and consistency, outperforming both Consensus and GPT-4o mini. Although LLMs are not positioned to replace clinicians, they may enhance evidence-based care following rigorous validation [20]. These findings mirror our results, which similarly identify GPT-4o as the top-performing model, followed by GPT-4.

Beyond diagnostic reasoning, Ayers et al. showed that ChatGPT-generated responses to patient questions were preferred over physician responses in 78.6% of cases and were rated superior in quality and empathy, indicating a potential role for AI assistants in improving patient communication and reducing clinician workload [29]. Moreover, the recent release of ChatGPT Health for real-world personal health applications underscores that LLM integration into clinical workflows has already begun, reinforcing the need for structured evaluation, safety monitoring and responsible deployment before widespread adoption [25].

Additionally, prior studies have demonstrated ChatGPT’s promising diagnostic potential and strong performance in clinical decision-making, particularly for final diagnosis, with performance improving as more clinical information becomes available [30,31]. Interestingly, Schmidt et al. showed that although ChatGPT achieves diagnostic accuracy comparable to that of medical residents, it remains susceptible to bias in patient histories, highlighting important limitations in clinical reasoning and reinforcing the need for cautious implementation and further investigation of AI bias [32].

The verbosity analysis adds a dimension not routinely reported in comparable studies. All models elaborated beyond the wording of the source guideline, but to very different degrees, ranging from 1.29-fold for Consensus Premium to 3.19-fold for GPT-4o. Elaboration is not inherently desirable: at the point of care, a concise, accurate answer may be more usable than an extended one, and additional text also creates additional surface for unsupported content. Longer answers accompanied higher ratings when the five systems were compared in aggregate, but an association across five aggregated points cannot show that length improves quality, and assessors’ exposure to the full answer may have produced a length halo effect on comprehensiveness ratings. Moreover, GPT-4 matched GPT-4o on accuracy while producing 20% fewer words, and Consensus Premium’s brevity was accompanied by omission rather than precision. Verbosity should therefore be regarded as a descriptive characteristic of model behaviour and a potential usability consideration, not as a proxy for quality. This is consistent with observations in other forms of digital health information: in the study of weight-management short videos by Du et al., popularity was only weakly related to independently rated quality, and duration contributed to quality prediction only beyond a threshold, underscoring that surface features cannot substitute for independent, multidimensional assessment of scientific quality and reliability [22]. More informative endpoints for future studies include the density of unsupported claims, the time clinicians need to correct an answer, actionability and whether an answer contains the information needed to reach the correct decision.

Taken together, our findings indicate that some LLMs can reproduce the content of the EKIT-Hip recommendations when asked context-free questions. They do not show that these systems perform at a level adequate for decision support in routine clinical practice, which would require evaluation with standardised patient cases containing diagnostic, treatment-selection, contraindication and risk-communication information, and comparison of clinicians’ decision accuracy, time and harm with and without model assistance. However, performance across the evaluated models was uneven, with GPT-4o and GPT-4 receiving the highest ratings. Beyond comparative performance, these findings highlight the importance of specialty-specific evaluation when translating AI into clinical practice, as meaningful implementation will depend on demonstrating utility for clinicians while ultimately improving patient outcomes. On the basis of these data, none of the evaluated systems should be used for unsupervised clinical decision-making.

Implementation of LLMs in clinical practice should therefore be grounded in evidence-based medicine and guided by established translational frameworks such as IDEAL, which provides a staged pathway (five stages, or six including the preclinical Stage 0) from preclinical evaluation and proof-of-concept testing through randomised assessment to long-term surveillance, and which has long informed the safe introduction of surgical innovation [33]. Given persistent concerns about safety, reliability and transparency as barriers to AI integration, Farrow et al. adapted these principles into an AI-specific five-stage framework spanning concept development to post-deployment model surveillance, offering a structured roadmap for responsible clinical implementation of AI in trauma and orthopaedics [34]. Within this framework, the present study corresponds to an early-stage, pre-clinical evaluation: it indicates that some models can reproduce guideline content in a single run of context-free questions, which is a necessary but not sufficient condition for clinical deployment. Clearly defined pathways for safe use, ongoing monitoring and escalation of care remain essential until model outputs have been validated prospectively in real-world orthopaedic practice.

### 4.1. Strengths and Limitations

The strengths of this study include the derivation of questions from five of the six thematic complexes of a current national S3 guideline, with item-level mapping to the reference recommendations (Table S1); blinded expert scoring with a predefined rubric; the inclusion of five widely used systems; and a statistical approach that accounts for the paired, question-clustered design.

Several limitations must nonetheless be acknowledged. First, the evaluation was based on a fixed set of 26 structured clinical questions derived exclusively from the 2021 DGOU hip OA guideline. Model performance may differ with alternative phrasing, broader orthopaedic scenarios or patient-specific contexts such as comorbidities or postoperative conditions. The findings therefore reflect performance within a defined guideline framework rather than the full complexity of routine clinical decision-making. The sample of 26 items per model also limits the precision of pairwise comparisons; several differences with medium effect sizes, including Consensus Premium versus Gemini Advanced, did not reach significance after correction for multiple testing and may represent type II error. In addition, the questions and reference answers were constructed by the investigators, which may introduce construct bias, and the sixth thematic complex (shared decision-making) was not covered.

Second, the analysis was restricted to text-based interactions and did not assess the ability of LLMs to interpret imaging, adapt to evolving clinical information or incorporate real-time data. Such capabilities are increasingly relevant in clinical practice and could influence model output once enabled within healthcare systems. The primary benchmark analysed a single response per question per system; a new chat session reduces context carry-over but does not address stochastic variation between repeated generations, prompt sensitivity or language effects, which therefore remain unquantified. This matters most where systems received similar ratings. GPT-4o and GPT-4 received different accuracy ratings for only 8 of the 26 questions (5 favouring GPT-4o and 3 favouring GPT-4); in a question-level bootstrap (10,000 resamples), GPT-4o had the highest mean accuracy in 76% of resamples and GPT-4 in 24%, and GPT-4o mini ranked above Gemini Advanced in 84%. By contrast, GPT-4o and GPT-4 occupied the two top positions in 99% of resamples, and Consensus Premium ranked last in more than 99%. Because this bootstrap reflects only the sampling of questions, run-to-run variation of the models would add further uncertainty; the order between GPT-4o and GPT-4, and between GPT-4o mini and Gemini Advanced, should therefore not be regarded as established, whereas the separation of the leading systems from Consensus Premium is more robust.

Third, although scoring was performed by three board-certified orthopaedic surgeons using blinded assessments and predefined criteria, all expert evaluation carries inherent subjectivity. This is a major methodological limitation: the assessors’ individual ratings were not recorded before consensus was reached, so inter-rater reliability cannot be quantified and the reproducibility of the ratings is unknown. Despite blinding, answer structure, tone and citation style may have revealed the source system, and blinding success was not assessed.

Fourth, data were collected over approximately 15 weeks through consumer interfaces whose underlying models may have been updated without notice, and several of the evaluated versions have since been retired; version drift within the collection period cannot be excluded. LLMs are continuously updated, and their performance may change in ways that a single cross-sectional analysis cannot capture. The results therefore describe model performance at the time of testing rather than long-term capability. Fifth, the study relied on a single national guideline. Although the 2021 DGOU recommendations reflect current evidence-based care within Germany, guideline updates or emerging evidence may shift therapeutic standards over time, and periodic reassessment will be necessary to ensure continued alignment between model-generated recommendations and contemporary practice.

Sixth, the comparators were products rather than isolated models: Consensus Premium and Gemini Advanced incorporate retrieval, citation and interface policies that differ from those of ChatGPT, so differences between systems reflect entire product workflows and cannot be attributed to the underlying language models alone. Errors were captured only through the ordinal accuracy rating; no item-level classification of error type, detectability or potential for harm was performed. Finally, no clinical endpoints were assessed. The study contained no patient vignettes, clinician–model interaction, decision change, workflow assessment, patient outcomes or prospective safety endpoints, and it therefore cannot establish clinical decision-support effectiveness or safety.

### 4.2. Future Directions

Future research should extend beyond static text-based guideline queries and evaluate LLM performance in more clinically representative scenarios, including standardised patient cases containing diagnostic, treatment-selection, contraindication and risk-communication information, multimorbidity, longitudinal management decisions and shared decision-making conversations, ideally in clinician-in-the-loop designs that compare clinicians’ decision accuracy, time and harm with versus without model assistance and that use frozen, versioned models with repeated runs. Validation against updated guideline iterations and current literature will also be required to ensure that responses do not become outdated as training corpora age. Studies should additionally quantify output stability across repeated queries and report inter-rater reliability for expert scoring.

In addition, real-world implementation studies are needed to assess usability and reliability when LLMs are used by frontline clinicians, residents and medical students in real time. Integration with electronic health records and clinical workflows, already under way in systems such as ChatGPT Health, raises important questions regarding data privacy, medico-legal responsibility and clinical accountability. Finally, comparative evaluations across subspecialties, languages and healthcare systems will be essential to determine whether the trends observed in hip OA generalise across orthopaedic care.

## 5. Conclusions

In a single-run benchmark of 26 context-free questions derived from five of the six thematic complexes of the 2021 EKIT-Hip S3 guideline, GPT-4o and GPT-4 produced the answers rated most accurate and comprehensive, followed by GPT-4o mini, Gemini Advanced and Consensus Premium. No major errors were observed for the ChatGPT variants in this sample, but with 26 responses per system this does not exclude a clinically relevant error rate and does not demonstrate that any system is safe. The study measured guideline-concordant answer quality; it did not evaluate clinical decision-support effectiveness, clinician–model interaction, workflow, patient outcomes or safety endpoints, and its data do not support unsupervised clinical use of any of the evaluated systems. Evaluation with standardised patient cases, repeated and version-controlled model runs, clinician-in-the-loop designs and prospective safety endpoints is required before LLM-based decision support for hip OA can be considered.

## Figures and Tables

**Figure 1:**
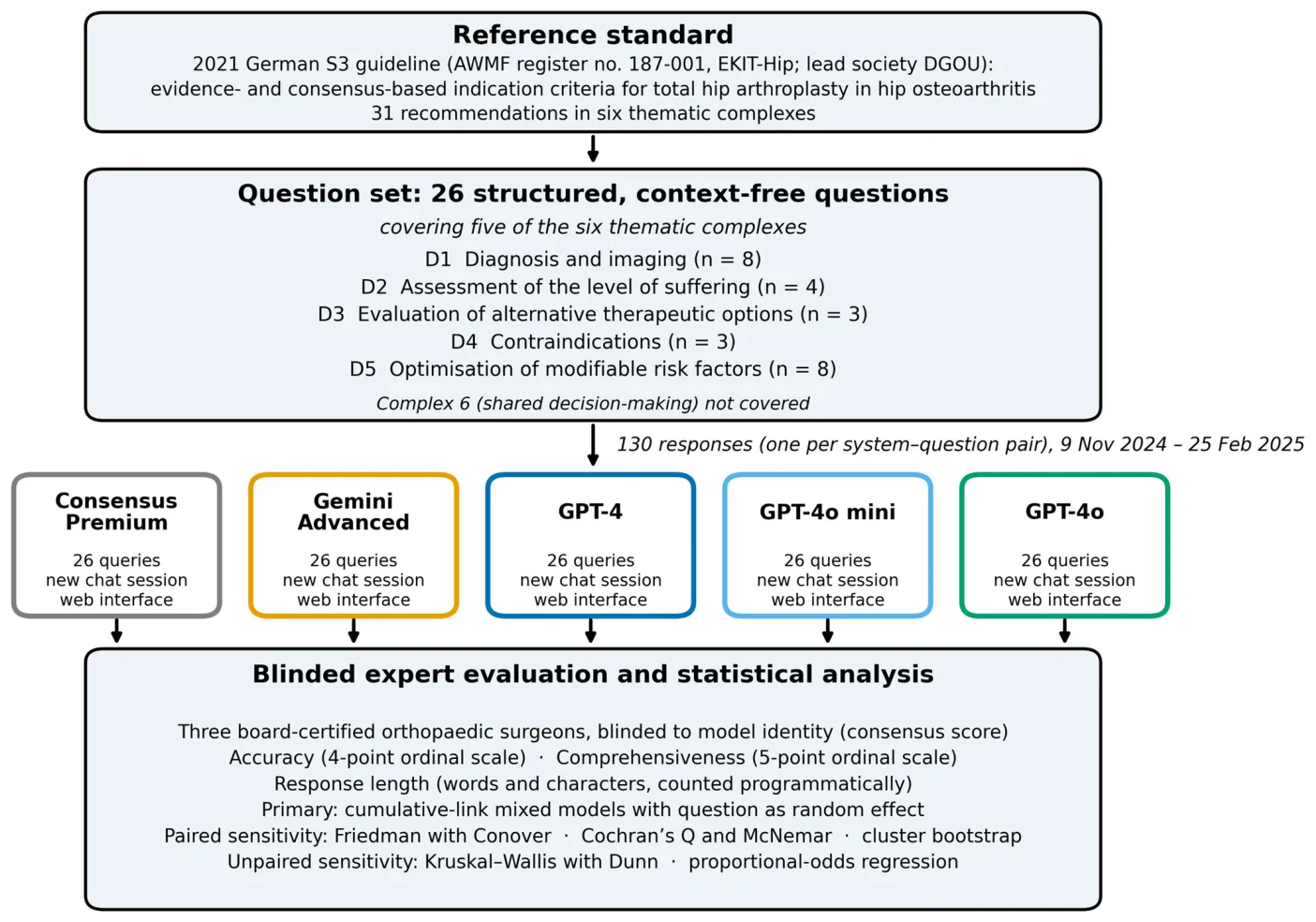
Overview of the study workflow. Twenty-six structured clinical questions, covering five of the six thematic complexes of the 2021 EKIT-Hip S3 guideline on indication criteria for total hip arthroplasty, were submitted to five large language models in newly initiated chat sessions, generating 130 responses. Responses were anonymised and independently evaluated by three board-certified orthopaedic surgeons blinded to model identity for accuracy and comprehensiveness; response length was counted programmatically.

**Figure 2:**
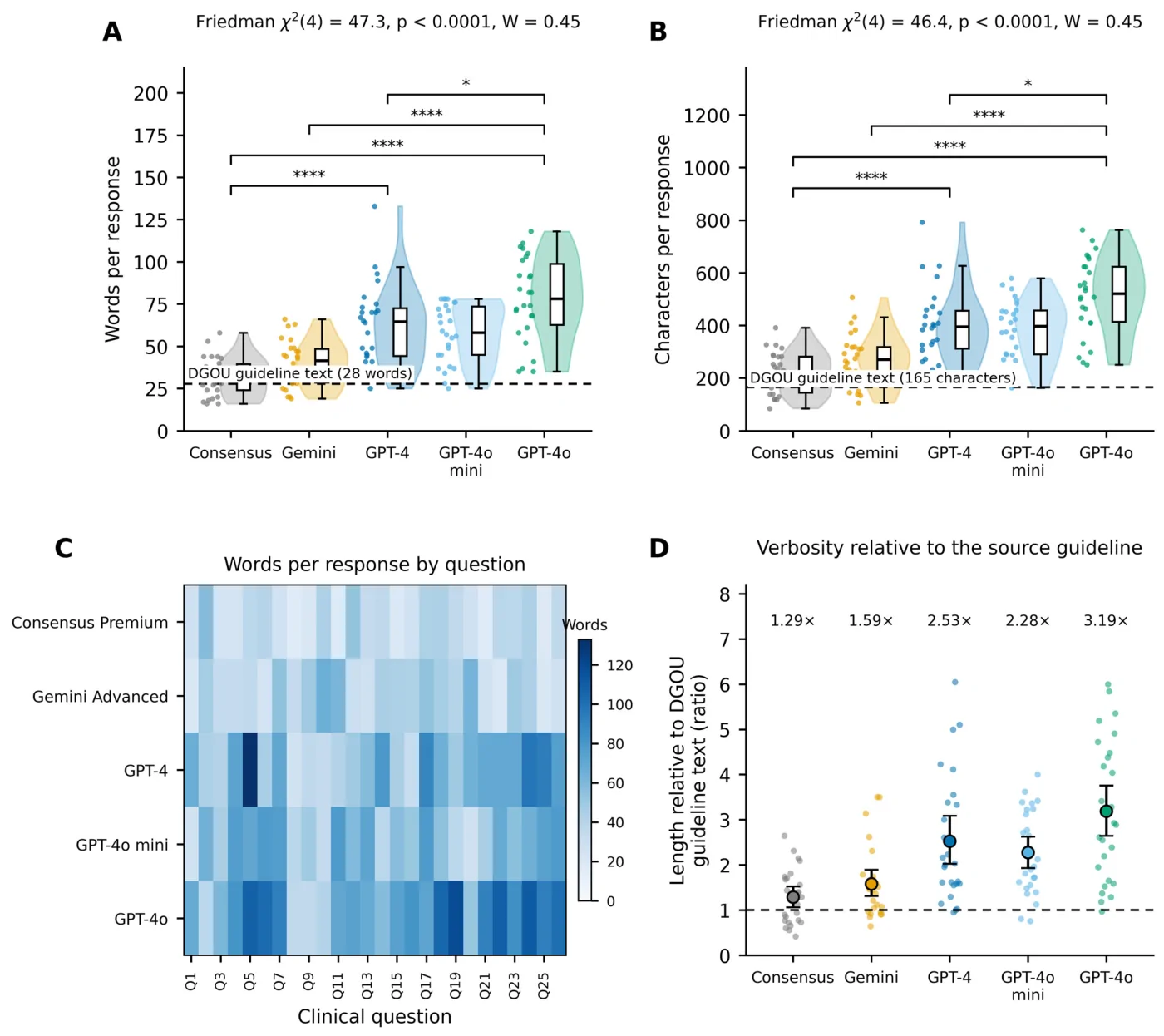
Response length across large language models for 26 guideline-based clinical questions on hip osteoarthritis. (**A**) Words per response and (**B**) characters per response, shown as individual question-level values (left), kernel density estimates and box plots (median, interquartile range and 1.5× interquartile range whiskers). The dashed line indicates the mean length of the corresponding DGOU guideline text. (**C**) Heat map of word counts for every model and every question. (**D**) Response length expressed as a ratio to the corresponding guideline text; large symbols denote the mean with bootstrap 95% confidence interval, and the dashed line marks parity with the guideline. Global comparisons used the Friedman test; pairwise comparisons used Conover’s post hoc test with Holm correction (* *p* < 0.05; **** *p* < 0.0001).

**Figure 3:**
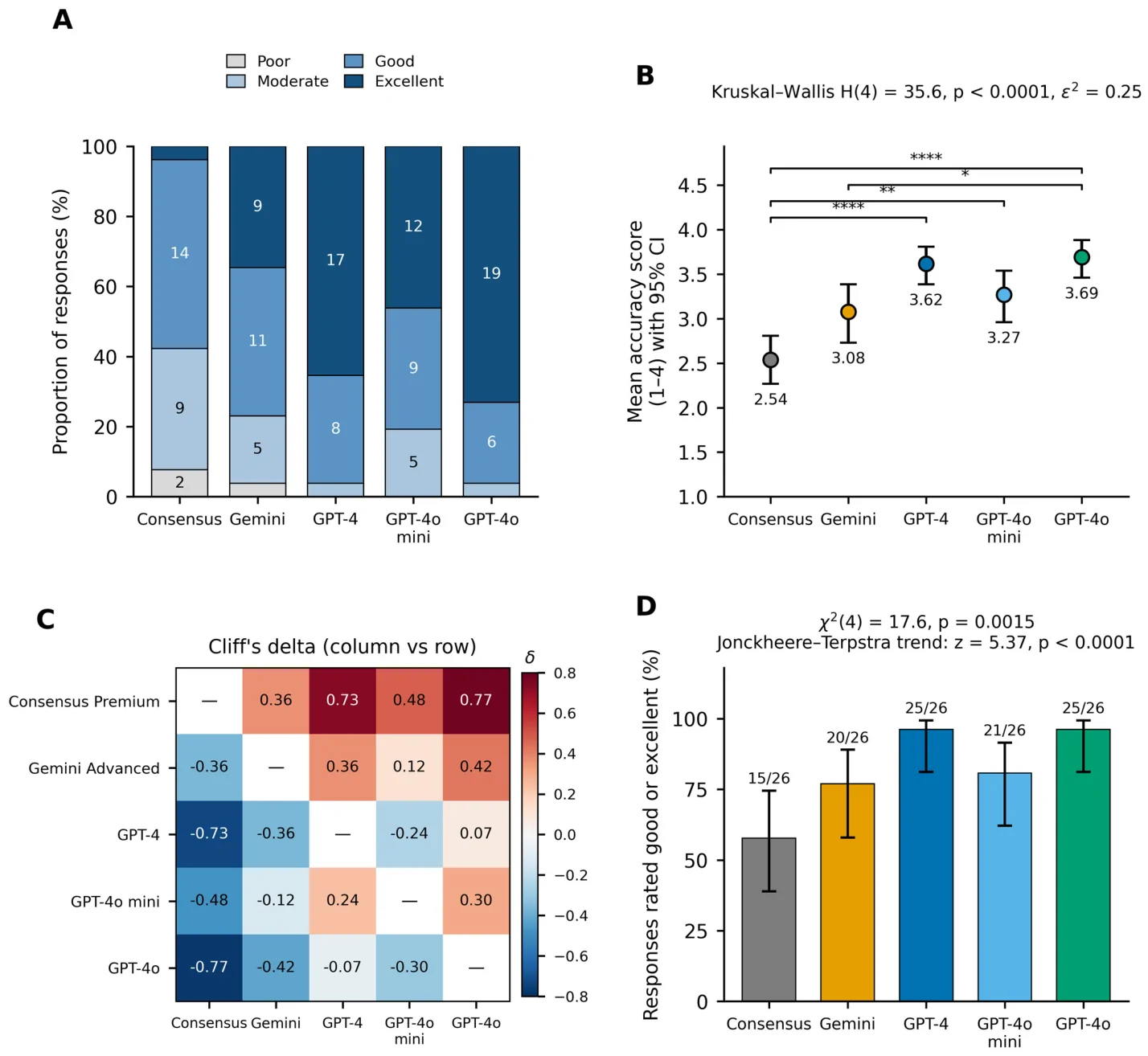
Accuracy of large language model responses assessed against the 2021 DGOU hip osteoarthritis guideline. (**A**) Distribution of accuracy ratings (poor, moderate, good, excellent); numbers within bars are response counts out of 26. (**B**) Mean accuracy score with bootstrap 95% confidence interval; brackets show significant pairwise differences from Dunn’s test with Holm correction. (**C**) Matrix of Cliff’s delta effect sizes; a positive value indicates that the column model outperformed the row model. (**D**) Proportion of responses rated good or excellent with 95% Wilson confidence intervals. Significance: * *p* < 0.05; ** *p* < 0.01; **** *p* < 0.0001. Brackets and effect sizes derive from the unpaired sensitivity analysis.

**Figure 4:**
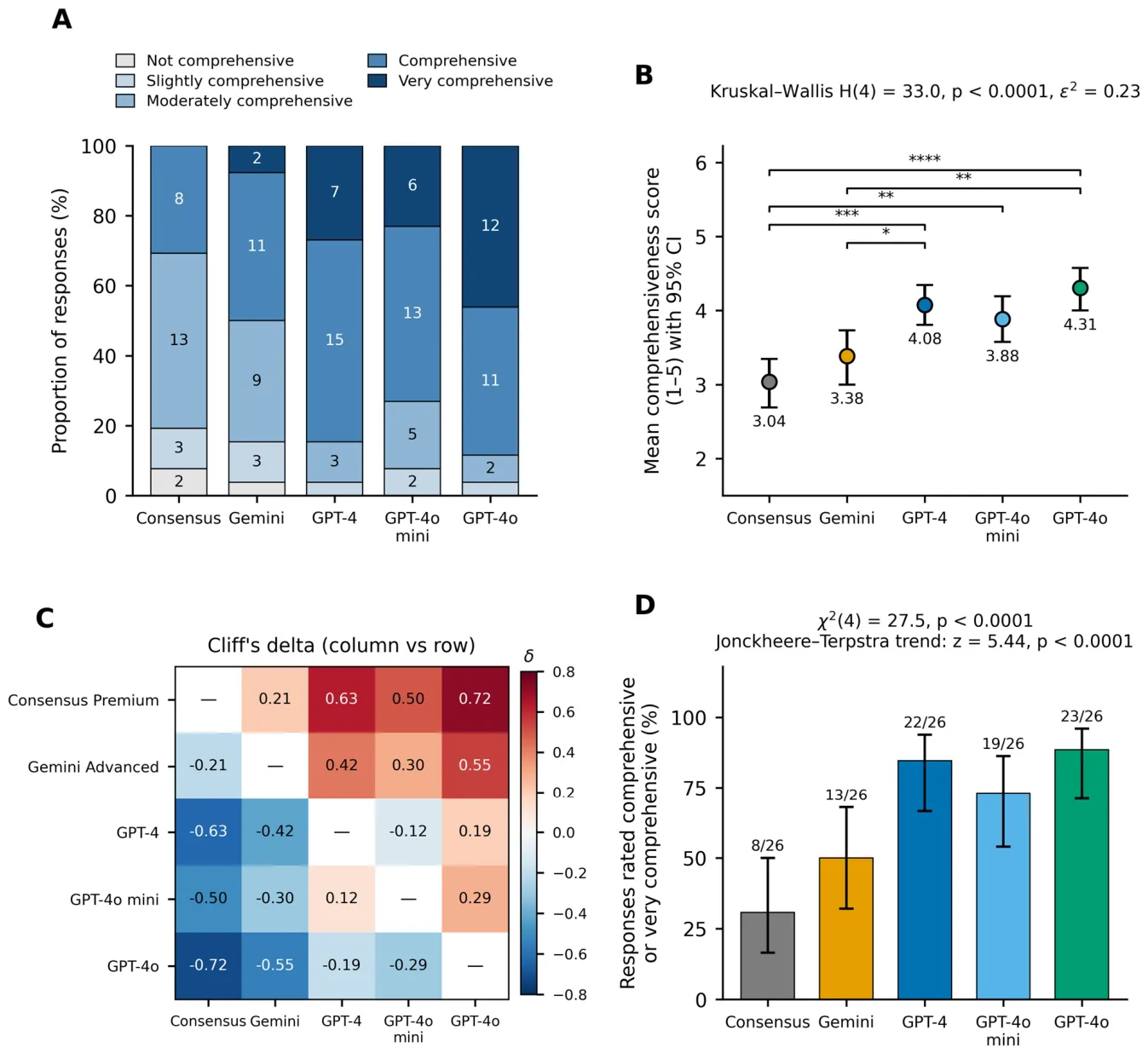
Comprehensiveness of large language model responses assessed against the 2021 DGOU hip osteoarthritis guideline. (**A**) Distribution of comprehensiveness ratings (not, slightly, moderately, comprehensive, very comprehensive); numbers within bars are response counts out of 26. (**B**) Mean comprehensiveness score with bootstrap 95% confidence interval; brackets show significant pairwise differences from Dunn’s test with Holm correction. (**C**) Matrix of Cliff’s delta effect sizes; a positive value indicates that the column model outperformed the row model. (**D**) Proportion of responses rated comprehensive or very comprehensive with 95% Wilson confidence intervals. Significance: * *p* < 0.05; ** *p* < 0.01; *** *p* < 0.001; **** *p* < 0.0001. Brackets and effect sizes derive from the unpaired sensitivity analysis.

**Figure 5:**
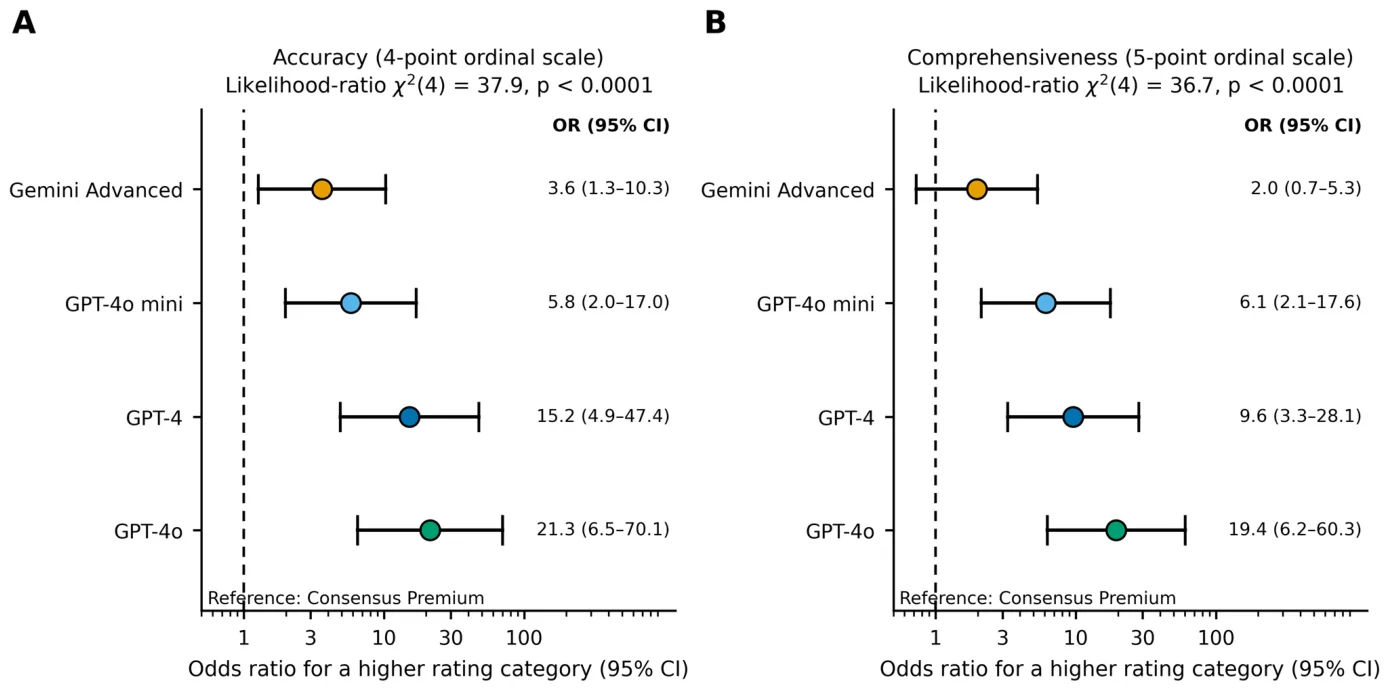
Proportional-odds ordinal logistic regression of (**A**) accuracy and (**B**) comprehensiveness ratings, with Consensus Premium as the reference category. Symbols show odds ratios for receiving a higher rating category and horizontal lines show 95% confidence intervals on a logarithmic scale. The dashed vertical line marks an odds ratio of 1.

**Figure 6:**
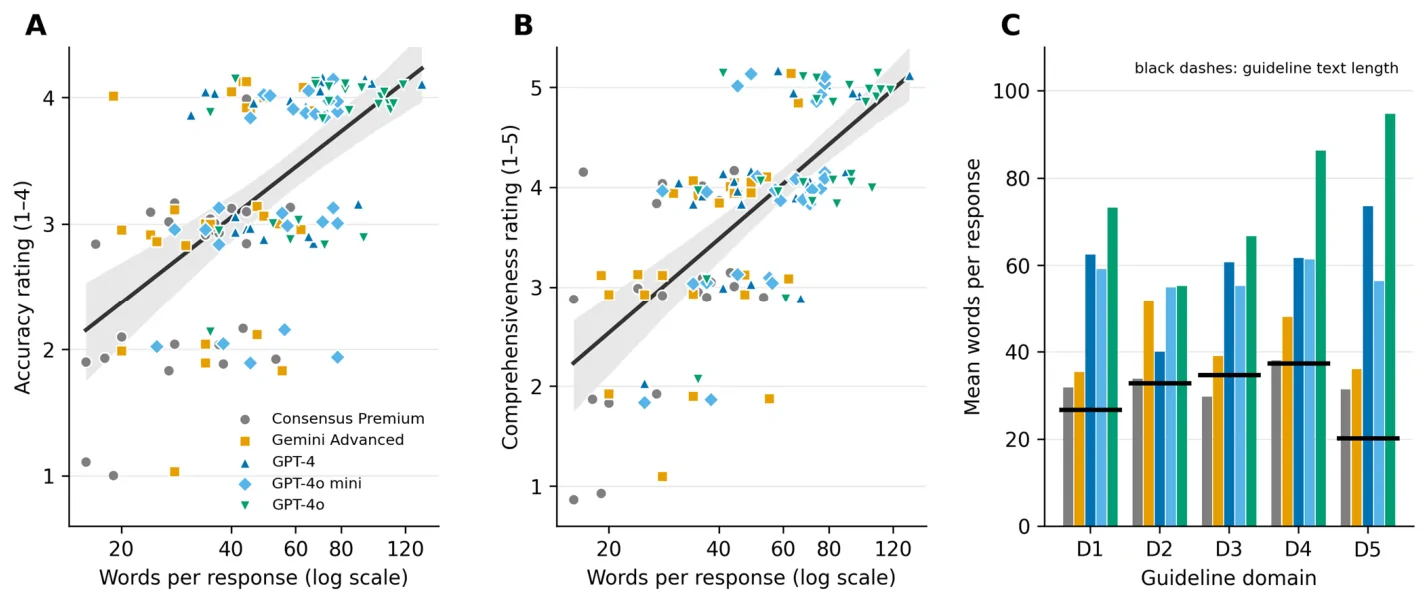
Response length and rated quality (exploratory). (**A**) Accuracy and (**B**) comprehensiveness ratings versus words per response for all 130 responses (question level; vertical jitter added for visibility), with the linear fit of rating on log word count and question-level cluster-bootstrap 95% confidence bands (2000 resamples). (**C**) Mean words per response by guideline domain (D1, diagnosis and imaging; D2, assessment of the level of suffering; D3, evaluation of alternative therapeutic options; D4, contraindications; D5, optimisation of modifiable risk factors); black dashes indicate the corresponding DGOU guideline text length; bars are coloured by system as in panels (**A**,**B**): grey, Consensus Premium; orange, Gemini Advanced; dark blue, GPT-4; light blue, GPT-4o mini; green, GPT-4o.

**Table 1:** Composition of the 26-item question set and its mapping to the 2021 DGOU hip osteoarthritis guideline.

Domain	Guideline Recommendation No.	Questions	n	Clinical Focus
D1—Diagnosis and imaging	4.1.1–4.1.8	Q1–Q8	8	History, clinical examination, imaging strategy and diagnostic work-up
D2—Assessment of the level of suffering	4.2.1–4.2.4	Q9–Q12	4	Pain burden, functional limitation and patient-reported impact
D3—Evaluation of alternative therapeutic options	4.3.1–4.3.3	Q13–Q15	3	Non-operative and pharmacological alternatives before arthroplasty
D4—Contraindications	4.4.1–4.4.4	Q16–Q18	3	Absolute and relative contraindications to surgical treatment
D5—Optimisation of modifiable risk factors	4.5.1–4.5.8	Q19–Q26	8	Weight, smoking, diabetes, anaemia and peri-operative optimisation
Total	—	Q1–Q26	26	—

DGOU, German Society for Orthopaedics and Trauma Surgery. Each question was mapped to one numbered recommendation of the EKIT-Hip guideline (AWMF 187-001). The sixth thematic complex (shared decision-making) was not covered. The recommendation number for each question is listed in Supplementary Table S1, and the full text of each recommendation with its grade and consensus strength in Supplementary Table S5.

**Table 2:** Response length of the five evaluated large language models, benchmarked against the corresponding DGOU guideline text.

Model	Words, Mean ± SD	Words, Median (IQR)	Words, Range	Characters, Mean ± SD	Characters, Median (IQR)	Ratio to Guideline Text *
DGOU guideline text	27.8 ± 10.0	25.0 (21.2–31.0)	14–56	165.3 ± 69.1	146.5 (113.8–191.2)	1.00 (reference)
Consensus Premium	32.4 ± 11.5	34.5 (24.0–39.5)	16–58	217.2 ± 80.4	224.5 (145.0–281.8)	1.29 ± 0.60
Gemini Advanced	40.0 ± 13.7	41.5 (28.5–48.5)	19–66	271.5 ± 99.4	270.0 (215.5–318.5)	1.59 ± 0.80
GPT-4	62.1 ± 24.3	64.5 (44.2–72.5)	25–133	400.3 ± 141.4	394.5 (311.8–456.0)	2.53 ± 1.41
GPT-4o mini	57.5 ± 17.2	58.0 (45.0–73.5)	25–78	384.5 ± 115.9	397.0 (290.8–457.0)	2.28 ± 0.93
GPT-4o	77.8 ± 24.9	78.0 (62.8–98.8)	35–118	512.1 ± 147.3	520.5 (414.5–623.5)	3.19 ± 1.57

Values are derived from 26 clinical questions per model. IQR, interquartile range; SD, standard deviation. * Ratio of model response length to the corresponding DGOU guideline text, computed per question and then averaged. Global comparison by Friedman test: words χ^2^(4) = 47.3, *p* < 0.0001; characters χ^2^(4) = 46.4, *p* < 0.0001.

**Table 3:** Distribution of accuracy ratings and summary statistics for the five evaluated large language models.

Model	Poor n (%)	Moderate n (%)	Good n (%)	Excellent n (%)	Mean ± SD	Median (IQR)	Mean Rank	Good or Excellent, n (%)
Consensus Premium	2 (7.7)	9 (34.6)	14 (53.8)	1 (3.8)	2.54 ± 0.71	3 (2–3)	35.0	15 (57.7)
Gemini Advanced	1 (3.8)	5 (19.2)	11 (42.3)	9 (34.6)	3.08 ± 0.84	3 (3–4)	58.4	20 (76.9)
GPT-4	0 (0.0)	1 (3.8)	8 (30.8)	17 (65.4)	3.62 ± 0.57	4 (3–4)	81.8	25 (96.2)
GPT-4o mini	0 (0.0)	5 (19.2)	9 (34.6)	12 (46.2)	3.27 ± 0.78	3 (3–4)	66.3	21 (80.8)
GPT-4o	0 (0.0)	1 (3.8)	6 (23.1)	19 (73.1)	3.69 ± 0.55	4 (3–4)	85.9	25 (96.2)

Accuracy was rated on a four-point ordinal scale: 1 = poor, 2 = moderate, 3 = good, 4 = excellent. Mean rank derives from the Kruskal–Wallis analysis across all 130 responses (H(4) = 35.6, *p* < 0.0001, ε^2^ = 0.25). IQR, interquartile range; SD, standard deviation.

**Table 4:** Distribution of comprehensiveness ratings and summary statistics for the five evaluated large language models.

Model	Not Comprehensive n (%)	Slightly n (%)	Moderately n (%)	Comprehensive n (%)	Very Comprehensive n (%)	Mean ± SD	Median (IQR)	Mean Rank	Comprehensive or Very, n (%)
Consensus Premium	2 (7.7)	3 (11.5)	13 (50.0)	8 (30.8)	0 (0.0)	3.04 ± 0.87	3.0 (3.0–4.0)	38.8	8 (30.8)
Gemini Advanced	1 (3.8)	3 (11.5)	9 (34.6)	11 (42.3)	2 (7.7)	3.38 ± 0.94	3.5 (3.0–4.0)	51.8	13 (50.0)
GPT-4	0 (0.0)	1 (3.8)	3 (11.5)	15 (57.7)	7 (26.9)	4.08 ± 0.74	4.0 (4.0–4.8)	78.2	22 (84.6)
GPT-4o mini	0 (0.0)	2 (7.7)	5 (19.2)	13 (50.0)	6 (23.1)	3.88 ± 0.86	4.0 (3.2–4.0)	70.6	19 (73.1)
GPT-4o	0 (0.0)	1 (3.8)	2 (7.7)	11 (42.3)	12 (46.2)	4.31 ± 0.79	4.0 (4.0–5.0)	88.1	23 (88.5)

Comprehensiveness was rated on a five-point ordinal scale: 1 = not comprehensive, 2 = slightly comprehensive, 3 = moderately comprehensive, 4 = comprehensive, 5 = very comprehensive. Mean rank derives from the Kruskal–Wallis analysis across all 130 responses (H(4) = 33.0, *p* < 0.0001, ε^2^ = 0.23).

**Table 5:** Pairwise between-model comparisons for accuracy, comprehensiveness and response length. Accuracy and comprehensiveness: unpaired sensitivity analysis (see Section 3.4 for the paired primary analysis).

Comparison	Accuracy, Adjusted *p*	Accuracy, Cliff’s δ (Magnitude)	Comprehensiveness, Adjusted *p*	Comprehensiveness, Cliff’s δ (Magnitude)	Words, Adjusted *p*	Characters, Adjusted *p*
Consensus Premium vs. Gemini Advanced	0.093	0.36 (medium)	0.561	0.21 (small)	0.129	0.046
Consensus Premium vs. GPT-4	<0.0001	0.73 (large)	0.0006	0.63 (large)	<0.0001	<0.0001
Consensus Premium vs. GPT-4o mini	0.010	0.48 (large)	0.009	0.50 (large)	<0.0001	<0.0001
Consensus Premium vs. GPT-4o	<0.0001	0.77 (large)	<0.0001	0.72 (large)	<0.0001	<0.0001
Gemini Advanced vs. GPT-4	0.093	0.36 (medium)	0.043	0.42 (medium)	0.003	0.014
Gemini Advanced vs. GPT-4o mini	0.828	0.12 (negligible)	0.281	0.30 (small)	0.001	0.014
Gemini Advanced vs. GPT-4o	0.031	0.42 (medium)	0.002	0.55 (large)	<0.0001	<0.0001
GPT-4 vs. GPT-4o mini	0.327	−0.24 (small)	0.628	−0.12 (negligible)	0.816	1.000
GPT-4 vs. GPT-4o	0.828	0.07 (negligible)	0.628	0.19 (small)	0.039	0.027
GPT-4o mini vs. GPT-4o	0.172	0.30 (small)	0.299	0.29 (small)	0.055	0.027

Accuracy and comprehensiveness were compared using Dunn’s test with Holm adjustment; words and characters were compared using Conover’s post hoc test for the Friedman analysis with Holm adjustment. Cliff’s delta (δ) thresholds: |δ| < 0.147 negligible; 0.147–0.33 small; 0.33–0.474 medium; >0.474 large. A positive δ indicates that the second-named model outperformed the first.

**Table 6:** Proportional-odds ordinal logistic regression of accuracy and comprehensiveness ratings. Model without random effects (unpaired sensitivity analysis); the primary cumulative-link mixed-model estimates are reported in Section 3.4 and Supplementary Table S3.

Model	Accuracy OR (95% CI)	Accuracy *p*	Comprehensiveness OR (95% CI)	Comprehensiveness *p*
Consensus Premium	1.00 (reference)	—	1.00 (reference)	—
Gemini Advanced	3.62 (1.27–10.29)	0.016	1.97 (0.73–5.32)	0.184
GPT-4	15.22 (4.88–47.44)	<0.0001	9.58 (3.26–28.10)	<0.0001
GPT-4o mini	5.81 (1.99–17.00)	0.001	6.10 (2.11–17.64)	0.0008
GPT-4o	21.33 (6.49–70.06)	<0.0001	19.41 (6.25–60.29)	<0.0001

Proportional-odds ordinal logistic regression with Consensus Premium as the reference category. An odds ratio above 1 indicates higher odds of being assigned a higher rating category. Accuracy model: likelihood-ratio χ^2^(4) = 37.9, *p* < 0.0001, McFadden’s pseudo-R^2^ = 0.13. Comprehensiveness model: likelihood-ratio χ^2^(4) = 36.7, *p* < 0.0001, pseudo-R^2^ = 0.11. CI, confidence interval; OR, odds ratio.

## Data Availability

The question-level consensus ratings and response lengths for all 130 responses (Data S1), the mapping of each question to its guideline recommendation (Table S1), the full text of the guideline recommendations with their grades and consensus strength (Table S5) and the full analysis code (Code S1) are provided as Supplementary Material. The verbatim model responses are available from the corresponding author on reasonable request.

## Supplementary Materials

The following supporting information can be downloaded at: https://www.mdpi.com/article/10.3390/jcm15207944/s1, Table S1: Word and character counts for every clinical question, every model and the corresponding DGOU guideline text; Table S2: Response length by guideline domain. Table S3: Cumulative-link mixed-model estimates, including sensitivity models with domain; Table S4: Paired pairwise comparisons (mixed-model contrasts and Conover post hoc tests); Table S5: Guideline recommendations with grade of recommendation, level of evidence, consensus strength, English translation and corresponding question; Data S1: Question-level consensus ratings and response lengths for all 130 responses; Code S1: Analysis code.

## Abbreviations

AI	Artificial intelligence
CI	Confidence interval
DGOU	Deutsche Gesellschaft für Orthopädie und Unfallchirurgie (German Society for Orthopaedics and Trauma Surgery)
D5	Domain 5—Optimisation of modifiable risk factors
D2	Domain 2—Assessment of the patient’s level of suffering
GPT-4	Generative Pre-trained Transformer 4
GPT-4o	GPT-4o (OpenAI model designation)
GPT-4o mini	GPT-4o mini (OpenAI model designation)
IDEAL	Idea, Development, Exploration, Assessment, Long-term study
IQR	Interquartile range
LLM/LLMs	Large language model/Large language models
OA	Osteoarthritis
OR	Odds ratio
USMLE	United States Medical Licensing Examination
STROBE	Strengthening the Reporting of Observational Studies in Epidemiology
ε^2^	Epsilon-squared effect size
δ	Cliff’s delta effect size
W	Kendall’s W
χ^2^	Chi-square statistic
R^2^	Coefficient of determination; McFadden’s pseudo-R^2^ is used in the text
AWMF	Association of the Scientific Medical Societies in Germany
CLMM	Cumulative-link mixed model
EKIT-Hip	Evidence- and consensus-based indication criteria for total hip arthroplasty in hip osteoarthritis (S3 guideline)
ICC	Intraclass correlation coefficient
