# Supplementary material for: Guideline-Concordant Answer Quality of Five Large Language Models on Indication Criteria for Total Hip Arthroplasty in Hip Osteoarthritis: A Single-Run Benchmark Against the 2021 German S3 Guideline (EKIT-Hip)
Source: J Clin Med. 2026 Oct 14;15(20):7944. doi: 10.3390/jcm15207944 (PMC13706208; doi:10.3390/jcm15207944)
Supplement: Supplementary file 1 [file jcm-15-07944-s001.zip › jcm-4566380 - Code_S1.pdf]

Code S1. R analysis code (file originally named Code_S1_analysis.R).

```
###############################################################################
# Code S1. Analysis code
#
# Guideline-Concordant Answer Quality of Five Large Language Models on
# Indication Criteria for Total Hip Arthroplasty in Hip Osteoarthritis:
# A Single-Run Benchmark Against the 2021 German S3 Guideline (EKIT-Hip)
#
# Input
#   Data_S1_question_level_ratings.csv   130 rows (26 questions x 5 systems):
#     question_id, domain, recommendation_no, model, accuracy (1-4),
#     comprehensiveness (1-5), words, characters, guideline_words,
#     guideline_characters
#   Table_S5_guideline_recommendations.csv   (optional) 26 rows
#
# Output (written to ./output)
#   Supplementary_Tables_S1-S5.xlsx  Tables S1-S5, one sheet each
#   Table_S1.csv ... Table_S4.csv    the same tables as plain CSV
#   analysis_log.txt                 all test statistics reported in the paper
#   manuscript_check.csv             every reported value recomputed and
#                                    compared with the manuscript (PASS/FAIL)
#
# Software: R 4.3.3; ordinal 2023.12-4; lme4 1.1-35; openxlsx.
# Usage:    Rscript Code_S1_analysis.R [data_dir]
# Runtime:  about 10-20 min (2000 cluster-bootstrap refits of each CLMM).
#           Set N_BOOT_CLMM <- 200 for a quick run.
###############################################################################

suppressPackageStartupMessages({
  library(ordinal)
  library(lme4)
  library(openxlsx)
})

# UTF-8 locale so that en dashes and ± in table cells are written correctly
for (loc in c("en_US.UTF-8", "C.UTF-8", "English_United States.utf8"))
  if (nzchar(suppressWarnings(Sys.setlocale("LC_CTYPE", loc)))) break

args     <- commandArgs(trailingOnly = TRUE)
DATA_DIR <- if (length(args) >= 1) args[1] else "."
OUT_DIR  <- file.path(DATA_DIR, "output")
dir.create(OUT_DIR, showWarnings = FALSE)

N_BOOT_CLMM <- 2000    # question-level cluster bootstrap of CLMM odds ratios
N_BOOT_RANK <- 10000   # question-level bootstrap of the ranking of systems
N_BOOT_DIFF <- 2000    # cluster bootstrap of paired differences
SEED        <- 20241109
EQ_MARGIN   <- 0.5     # TOST equivalence margin (rating points)

MODELS  <- c("Consensus Premium", "Gemini Advanced", "GPT-4", "GPT-4o mini", "GPT-4o")
DOMAINS <- c(D1 = "Diagnosis and imaging",
             D2 = "Assessment of the level of suffering",
             D3 = "Evaluation of alternative therapeutic options",
             D4 = "Contraindications",
             D5 = "Optimisation of modifiable risk factors")

log_con <- file(file.path(OUT_DIR, "analysis_log.txt"), open = "wt")
say <- function(...) { txt <- sprintf(...); cat(txt, "\n"); cat(txt, "\n", file = log_con) }
hdr <- function(x) say("\n==== %s ====", x)

###############################################################################
# 1. Read and validate data
###############################################################################
d <- read.csv(file.path(DATA_DIR, "Data_S1_question_level_ratings.csv"),
              stringsAsFactors = FALSE, check.names = FALSE, encoding = "UTF-8")

stopifnot_msg <- function(ok, msg) if (!all(ok)) stop(msg, call. = FALSE)
need <- c("question_id", "domain", "recommendation_no", "model", "accuracy",
          "comprehensiveness", "words", "characters", "guideline_words",
          "guideline_characters")
stopifnot_msg(need %in% names(d), paste("Missing columns:", paste(setdiff(need, names(d)), collapse = ", ")))
stopifnot_msg(nrow(d) == 130, sprintf("Expected 130 rows, found %d", nrow(d)))
stopifnot_msg(d$model %in% MODELS, "Unknown model label(s) in column 'model'")
stopifnot_msg(!anyNA(d[, c("accuracy", "comprehensiveness", "words", "characters",
                           "guideline_words", "guideline_characters")]),
              "Empty cells in rating or length columns - fill in all 130 rows")
stopifnot_msg(d$accuracy %in% 1:4, "accuracy must be 1-4")
stopifnot_msg(d$comprehensiveness %in% 1:5, "comprehensiveness must be 1-5")
stopifnot_msg(all(table(d$question_id, d$model) == 1), "Each question must have exactly one row per model")
gl_chk <- tapply(d$guideline_words, d$question_id, function(x) length(unique(x)))
stopifnot_msg(all(gl_chk == 1), "guideline_words must be identical for the 5 rows of a question")
```

```
qnum       <- as.integer(sub("^Q", "", d$question_id))
d$question <- factor(d$question_id, levels = paste0("Q", sort(unique(qnum))))
d$model    <- factor(d$model, levels = MODELS)
d$domain   <- factor(d$domain, levels = names(DOMAINS))
d$acc_f    <- factor(d$accuracy, levels = 1:4, ordered = TRUE)
d$comp_f   <- factor(d$comprehensiveness, levels = 1:5, ordered = TRUE)
d$ratio    <- d$words / d$guideline_words
d$good     <- as.integer(d$accuracy >= 3)          # guideline-concordant
d$highcomp <- as.integer(d$comprehensiveness >= 4) # highly comprehensive
d$major    <- as.integer(d$accuracy == 1)          # poor = major error
d          <- d[order(d$question, d$model), ]

nQ <- nlevels(d$question); k <- length(MODELS)
wide <- function(var) {        # questions x models matrix
  m <- tapply(d[[var]], list(d$question, d$model), identity)
  m[, MODELS, drop = FALSE]
}
glw <- tapply(d$guideline_words, d$question, `[`, 1)
glc <- tapply(d$guideline_characters, d$question, `[`, 1)

###############################################################################
# 2. Helper functions
###############################################################################
pfmt <- function(p) ifelse(is.na(p), "NA", ifelse(p < 0.0001, "<0.0001", formatC(p, digits = 4, format =
"f")))
msd  <- function(x, dg = 1) sprintf(paste0("%.", dg, "f ± %.", dg, "f"), mean(x), sd(x))
miqr <- function(x, dg = 1) { q <- quantile(x, c(.5, .25, .75), type = 7)
  sprintf(paste0("%.", dg, "f (%.", dg, "f-%.", dg, "f)"), q[1], q[2], q[3]) }
pairs_list <- combn(MODELS, 2, simplify = FALSE)
pair_lab   <- sapply(pairs_list, paste, collapse = " vs. ")

# Kendall's W from the Friedman statistic
kendall_w <- function(chi2, n, k) chi2 / (n * (k - 1))

# Conover post hoc test after Friedman (Conover 1999), Holm-adjusted
conover_friedman <- function(M) {
  R  <- t(apply(M, 1, rank))
  b  <- nrow(M); kk <- ncol(M)
  Rj <- colSums(R); A1 <- sum(R^2)
  se <- sqrt(2 * (b * A1 - sum(Rj^2)) / ((b - 1) * (kk - 1)))
  df <- (b - 1) * (kk - 1)
  p  <- sapply(pairs_list, function(pr) {
    tt <- abs(Rj[pr[1]] - Rj[pr[2]]) / se
    2 * pt(tt, df, lower.tail = FALSE) })
  p.adjust(p, "holm")
}

# Dunn test after Kruskal-Wallis, Holm-adjusted
dunn_test <- function(y, g) {
  r <- rank(y); N <- length(y)
  ties <- table(y); tc <- sum(ties^3 - ties) / (12 * (N - 1))
  mr <- tapply(r, g, mean); n <- tapply(r, g, length)
  p <- sapply(pairs_list, function(pr) {
    z <- (mr[pr[1]] - mr[pr[2]]) / sqrt((N * (N + 1) / 12 - tc) * (1 / n[pr[1]] + 1 / n[pr[2]]))
    2 * pnorm(-abs(z)) })
  p.adjust(p, "holm")
}

# Cliff's delta; positive = second-named group outperforms the first
cliff <- function(x1, x2) mean(sign(outer(x2, x1, "-")))
cliff_mag <- function(dl) cut(abs(dl), c(-Inf, 0.147, 0.33, 0.474, Inf),
                              c("negligible", "small", "medium", "large"), right = FALSE)

# Cochran's Q
cochran_q <- function(B) {
  kk <- ncol(B); Cj <- colSums(B); Ri <- rowSums(B); Nt <- sum(B)
  Q <- (kk - 1) * (kk * sum(Cj^2) - Nt^2) / (kk * Nt - sum(Ri^2))
  c(Q = Q, df = kk - 1, p = pchisq(Q, kk - 1, lower.tail = FALSE))
}

# Exact McNemar test (binomial test on discordant pairs)
mcnemar_exact <- function(a, b) {
  n01 <- sum(a == 1 & b == 0); n10 <- sum(a == 0 & b == 1)
  if (n01 + n10 == 0) return(1)
  binom.test(n01, n01 + n10, 0.5)$p.value
}

wilson <- function(x, n) suppressWarnings(prop.test(x, n, correct = FALSE)$conf.int[1:2])
cp     <- function(x, n) binom.test(x, n)$conf.int[1:2]

# CLMM fitting and extraction of the system coefficients
# Adaptive Gauss-Hermite quadrature (10 nodes) for models with one random effect;
# the Laplace approximation is used for the model with two random effects.
```

```r
N_AGQ <- 10
fit_clmm <- function(form, data) {
  n_re <- length(lme4::findbars(form))
  clmm(form, data = data, link = "logit", Hess = TRUE, nAGQ = if (n_re > 1) 1 else N_AGQ)
}
sys_coef <- function(fit) {
  b <- fit$beta; nm <- paste0("model", MODELS[-1]); b[nm] }
sys_vcov <- function(fit) {
  nm <- paste0("model", MODELS[-1])
  V <- tryCatch(vcov(fit), error = function(e) NULL)
  if (is.null(V)) {
    say("  Note: Hessian not positive definite for %s; Wald CIs not available", deparse(formula(fit)))
    return(matrix(NA, length(nm), length(nm), dimnames = list(nm, nm))) }
  V[nm, nm] }

# Question-level cluster bootstrap: resample questions, relabel duplicates
boot_sample <- function(data) {
  qs <- sample(levels(data$question), replace = TRUE)
  out <- do.call(rbind, lapply(seq_along(qs), function(i) {
    s <- data[data$question == qs[i], ]; s$question <- paste0("b", i); s }))
  out$question <- factor(out$question); out
}

################################################################################
# 3. Response length (Section 3.1, Table 2, Table S1, Table S2)
################################################################################
set.seed(SEED)
hdr("3.1 Response length")
W <- wide("words"); C <- wide("characters"); RT <- wide("ratio")

fw <- friedman.test(W); fc <- friedman.test(C)
say("Words: Friedman chi2(%d) = %.1f, p = %s, Kendall's W = %.2f",
    fw$parameter, fw$statistic, pfmt(fw$p.value), kendall_w(fw$statistic, nQ, k))
say("Characters: Friedman chi2(%d) = %.1f, p = %s, Kendall's W = %.2f",
    fc$parameter, fc$statistic, pfmt(fc$p.value), kendall_w(fc$statistic, nQ, k))
con_w <- conover_friedman(W); con_c <- conover_friedman(C)
for (i in seq_along(pairs_list))
  say("  Conover (Holm) %-38s words p = %s; characters p = %s", pair_lab[i], pfmt(con_w[i]), pfmt(con_c[i]))

say("Guideline text: words %s (median %s, range %d-%d); characters %s (median %s)",
    msd(glw), miqr(glw), min(glw), max(glw), msd(glc), miqr(glc))
wil <- sapply(MODELS, function(m) suppressWarnings(wilcox.test(W[, m], glw, paired = TRUE, exact = FALSE,
correct = FALSE)$p.value))
for (m in MODELS) say("  %-18s words %s; ratio %s x; Wilcoxon vs guideline p = %s",
                      m, msd(W[, m]), msd(RT[, m], 2), pfmt(wil[m]))

# ---- Table S1: word and character counts per question --------------------
q_meta <- d[!duplicated(d$question), c("question", "domain", "recommendation_no", "guideline_words",
"guideline_characters")]
S1 <- data.frame(Question = as.character(q_meta$question),
                 Domain = as.character(q_meta$domain),
                 `Guideline recommendation no.` = q_meta$recommendation_no,
                 `DGOU guideline text, words` = q_meta$guideline_words,
                 `DGOU guideline text, characters` = q_meta$guideline_characters,
                 check.names = FALSE)
for (m in MODELS) {
  S1[[paste0(m, ", words")]]      <- W[as.character(S1$Question), m]
  S1[[paste0(m, ", characters")]] <- C[as.character(S1$Question), m]
}
S1_tot <- S1[1, ]; S1_tot[1, ] <- NA
S1_tot$Question <- "Mean ± SD"
for (cn in names(S1)[-(1:3)]) S1_tot[[cn]] <- msd(S1[[cn]])
S1_out <- rbind(transform(S1, check.names = FALSE), S1_tot)
names(S1_out) <- names(S1)

# ---- Table S2: response length by guideline domain ------------------------
S2 <- do.call(rbind, lapply(names(DOMAINS), function(dm) {
  qs  <- levels(droplevels(d$question[d$domain == dm]))
  Wd  <- W[qs, , drop = FALSE]; Cd <- C[qs, , drop = FALSE]
  fr  <- if (length(qs) >= 2) friedman.test(Wd) else NULL
  frc <- if (length(qs) >= 2) friedman.test(Cd) else NULL
  row <- data.frame(Domain = paste0(dm, "–", DOMAINS[dm]), `n questions` = length(qs),
                    `DGOU guideline text, words` = msd(glw[qs]),
                    `DGOU guideline text, characters` = msd(glc[qs]), check.names = FALSE)
  for (m in MODELS) row[[paste0(m, ", words")]] <- msd(Wd[, m])
  for (m in MODELS) row[[paste0(m, ", characters")]] <- msd(Cd[, m])
  for (m in MODELS) row[[paste0(m, ", ratio to guideline")]] <- msd(RT[qs, m], 2)
  row$`Friedman words, chi2 (p)` <- if (is.null(fr)) "NA" else sprintf("%.2f (%s)", fr$statistic,
pfmt(fr$p.value))
  row$`Friedman characters, chi2 (p)` <- if (is.null(frc)) "NA" else sprintf("%.2f (%s)", frc$statistic,
pfmt(frc$p.value))
  say("  %s: Friedman words chi2 = %.2f, p = %s; GPT-4o %s words vs guideline %s",
      dm, fr$statistic, pfmt(fr$p.value), msd(Wd[, "GPT-4o"]), msd(glw[qs]))
  row }))
```

```
###############################################################################
# 4. Accuracy and comprehensiveness: descriptive, paired and unpaired tests
###############################################################################
outcomes <- list(accuracy = list(var = "accuracy", fac = "acc_f", bin = "good", K = 4),
                 comprehensiveness = list(var = "comprehensiveness", fac = "comp_f", bin = "highcomp", K = 5))
res <- list()

for (oc in names(outcomes)) {
  o <- outcomes[[oc]]; hdr(paste("Outcome:", oc)); M <- wide(o$var)
  for (m in MODELS) {
    x <- M[, m]
    say("  %-18s mean %s; median (IQR) %s; counts %s; boot 95%% CI of mean %.2f-%.2f",
        m, msd(x, 2), miqr(x), paste(tabulate(x, o$K), collapse = "/"),
        quantile(replicate(N_BOOT_DIFF, mean(sample(x, replace = TRUE))), .025),
        quantile(replicate(N_BOOT_DIFF, mean(sample(x, replace = TRUE))), .975))
  }
  # paired: Friedman + Conover
  fr <- friedman.test(M)
  say("Friedman chi2(%d) = %.1f, p = %s, Kendall's W = %.2f", fr$parameter, fr$statistic,
      pfmt(fr$p.value), kendall_w(fr$statistic, nQ, k))
  con <- conover_friedman(M)
  # unpaired: Kruskal-Wallis + Dunn + Cliff's delta
  kw <- kruskal.test(d[[o$var]] ~ d$model)
  eps2 <- (unname(kw$statistic) - k + 1) / (nrow(d) - k)   # epsilon^2 = (H - k + 1)/(n - k)
  mean_rank <- tapply(rank(d[[o$var]]), d$model, mean)
  say("Kruskal-Wallis H(%d) = %.1f, p = %s, epsilon^2 = %.2f; mean ranks %s", kw$parameter,
      kw$statistic, pfmt(kw$p.value), eps2, paste(sprintf("%.1f", mean_rank), collapse = "/"))
  dunn <- dunn_test(d[[o$var]], d$model)
  cd   <- sapply(pairs_list, function(pr) cliff(M[, pr[1]], M[, pr[2]]))
  # dichotomised outcome
  B  <- wide(o$bin); cq <- cochran_q(B)
  ct <- suppressWarnings(chisq.test(table(d$model, d[[o$bin]])))
  say("Dichotomised (%s): counts %s; Cochran's Q = %.2f, df = %d, p = %s; unpaired chi2(%d) = %.1f, p = %s",
      o$bin, paste(colSums(B), collapse = "/"), cq["Q"], cq["df"], pfmt(cq["p"]),
      ct$parameter, ct$statistic, pfmt(ct$p.value))
  for (m in MODELS) { ci <- wilson(sum(B[, m]), nQ)
    say("    %-18s %d/26 (%.1f%%), Wilson 95%% CI %.1f-%.1f%%", m, sum(B[, m]), 100 * mean(B[, m]), 100 *
ci[1], 100 * ci[2]) }
  mcn <- p.adjust(sapply(pairs_list, function(pr) mcnemar_exact(B[, pr[1]], B[, pr[2]])), "holm")
  pdiff <- t(sapply(pairs_list, function(pr) {
    dd <- B[, pr[2]] - B[, pr[1]]
    bt <- replicate(N_BOOT_DIFF, mean(sample(dd, replace = TRUE)))
    c(mean(dd), quantile(bt, c(.025, .975))) }))
  # paired mean differences in ratings
  mdiff <- t(sapply(pairs_list, function(pr) {
    dd <- M[, pr[2]] - M[, pr[1]]
    bt <- replicate(N_BOOT_DIFF, mean(sample(dd, replace = TRUE)))
    c(mean(dd), quantile(bt, c(.025, .975))) }))
  res[[oc]] <- list(M = M, fr = fr, con = con, kw = kw, eps2 = eps2, dunn = dunn, cd = cd,
                    cq = cq, ct = ct, mcn = mcn, pdiff = pdiff, mdiff = mdiff, mean_rank = mean_rank)

  # TOST: GPT-4o minus GPT-4
  dd <- M[, "GPT-4o"] - M[, "GPT-4"]; se <- sd(dd) / sqrt(nQ); df <- nQ - 1
  ci95 <- mean(dd) + c(-1, 1) * qt(.975, df) * se
  ci90 <- mean(dd) + c(-1, 1) * qt(.95, df) * se
  p_tost <- max(pt((mean(dd) + EQ_MARGIN) / se, df, lower.tail = FALSE),
                pt((mean(dd) - EQ_MARGIN) / se, df))
  res[[oc]]$tost <- c(diff = mean(dd), ci95, ci90, p = p_tost)
  say("GPT-4o minus GPT-4: %.2f (95%% CI %.2f to %.2f; 90%% CI %.2f to %.2f); TOST (+/-%.1f) p = %s; ratings
differ for %d questions (%d favour GPT-4o, %d GPT-4)",
      mean(dd), ci95[1], ci95[2], ci90[1], ci90[2], EQ_MARGIN, pfmt(p_tost),
      sum(dd != 0), sum(dd > 0), sum(dd < 0))
}

# major errors
hdr("Major errors (accuracy = poor)")
Bm <- wide("major"); cqm <- cochran_q(Bm)
for (m in MODELS) { ci <- cp(sum(Bm[, m]), nQ)
  say("  %-18s %d/26 (%.1f%%), Clopper-Pearson 95%% CI %.1f-%.1f%%", m, sum(Bm[, m]),
      100 * mean(Bm[, m]), 100 * ci[1], 100 * ci[2]) }
say("Cochran's Q = %.2f, df = %d, p = %s", cqm["Q"], cqm["df"], pfmt(cqm["p"]))

###############################################################################
# 5. Cumulative-link mixed models (primary analysis; Section 3.4, Table S3)
###############################################################################
clmm_res <- list(); S3_rows <- list()
for (oc in names(outcomes)) {
  o <- outcomes[[oc]]; hdr(paste("CLMM:", oc))
  f1 <- as.formula(paste(o$fac, "~ model + (1|question)"))
  f0 <- as.formula(paste(o$fac, "~ 1 + (1|question)"))
  fD <- as.formula(paste(o$fac, "~ model + domain + (1|question)"))
  fR <- as.formula(paste(o$fac, "~ model + (1|domain) + (1|question)"))
  m1 <- fit_clmm(f1, d); m0 <- fit_clmm(f0, d)
  mD <- fit_clmm(fD, d); mR <- fit_clmm(fR, d)
```

```
  lr <- anova(m0, m1)
  vq <- as.numeric(VarCorr(m1)$question)
  say("LR chi2(%d) = %.1f, p = %s; question intercept variance %.2f (SD %.2f)",
      lr$df[2], lr$LR.stat[2], pfmt(lr$`Pr(>Chisq)`[2]), vq, sqrt(vq))

  # proportional-odds assumption: likelihood-ratio test of a partial proportional-odds
  # (nominal-effects) model against the proportional-odds model; question is entered as
  # a fixed effect because ordinal::clmm() does not support nominal effects
  po <- tryCatch({
    d2 <- d; d2$y <- d2[[o$fac]]
    a  <- clm(y ~ model + question, data = d2)
    b  <- clm(y ~ question, nominal = ~ model, data = d2)
    stat <- 2 * (as.numeric(logLik(b)) - as.numeric(logLik(a)))
    dfp  <- (o$K - 2) * (k - 1)
    c(stat = stat, df = dfp, p = pchisq(stat, dfp, lower.tail = FALSE))
  }, error = function(e) { say("  PO test failed: %s", conditionMessage(e)); c(stat = NA, df = NA, p = NA) })
  say("Proportional-odds test: chi2(%s) = %.2f, p = %s", po["df"], po["stat"], pfmt(po["p"]))

  b1 <- sys_coef(m1); se1 <- sqrt(diag(sys_vcov(m1)))
  zp <- 2 * pnorm(-abs(b1 / se1))

  # cluster bootstrap of ORs
  set.seed(SEED)
  bt <- matrix(NA, N_BOOT_CLMM, k - 1, dimnames = list(NULL, MODELS[-1]))
  for (i in seq_len(N_BOOT_CLMM)) {
    bt[i, ] <- tryCatch(sys_coef(clmm(f1, data = boot_sample(d), link = "logit", nAGQ = N_AGQ)),
                        error = function(e) rep(NA, k - 1))
  }
  n_ok <- sum(complete.cases(bt))
  bci  <- apply(bt, 2, quantile, c(.025, .975), na.rm = TRUE)
  say("Cluster bootstrap: %d/%d successful refits", n_ok, N_BOOT_CLMM)

  bD <- sys_coef(mD); seD <- sqrt(diag(sys_vcov(mD)))
  bR <- sys_coef(mR); seR <- sqrt(diag(sys_vcov(mR)))
  for (j in seq_along(b1)) {
    m <- MODELS[j + 1]
    say("  %-16s OR %.2f (Wald %.2f-%.2f; boot %.2f-%.2f), p = %s | +domain fixed OR %.2f | +domain random
OR %.2f",
        m, exp(b1[j]), exp(b1[j] - 1.96 * se1[j]), exp(b1[j] + 1.96 * se1[j]),
        exp(bci[1, j]), exp(bci[2, j]), pfmt(zp[j]), exp(bD[j]), exp(bR[j]))
    S3_rows[[length(S3_rows) + 1]] <- data.frame(
      Outcome = oc, System = m,
      `Primary CLMM: OR (Wald 95% CI)` = sprintf("%.2f (%.2f–%.2f)", exp(b1[j]), exp(b1[j] - 1.96 * se1[j]),
exp(b1[j] + 1.96 * se1[j])),
      `Primary CLMM: cluster-bootstrap 95% CI` = sprintf("%.2f–%.2f", exp(bci[1, j]), exp(bci[2, j])),
      `Primary CLMM: p` = pfmt(zp[j]),
      `+ Domain (fixed): OR (95% CI)` = sprintf("%.2f (%.2f–%.2f)", exp(bD[j]), exp(bD[j] - 1.96 * seD[j]),
exp(bD[j] + 1.96 * seD[j])),
      `+ Domain (random): OR (95% CI)` = sprintf("%.2f (%.2f–%.2f)", exp(bR[j]), exp(bR[j] - 1.96 * seR[j]),
exp(bR[j] + 1.96 * seR[j])),
      check.names = FALSE)
  }
  S3_rows[[length(S3_rows) + 1]] <- data.frame(
    Outcome = oc, System = "Model statistics",
    `Primary CLMM: OR (Wald 95% CI)` = sprintf("LR chi2(%d) = %.1f, p %s", lr$df[2], lr$LR.stat[2],
pfmt(lr$`Pr(>Chisq)`[2])),
    `Primary CLMM: cluster-bootstrap 95% CI` = sprintf("%d/%d refits", n_ok, N_BOOT_CLMM),
    `Primary CLMM: p` = sprintf("Question variance %.2f (SD %.2f)", vq, sqrt(vq)),
    `+ Domain (fixed): OR (95% CI)` = sprintf("PO test chi2(%s) = %.2f, p = %s", po["df"], po["stat"],
pfmt(po["p"])),
    `+ Domain (random): OR (95% CI)` = sprintf("Domain variance %.2f", as.numeric(VarCorr(mR)$domain)),
    check.names = FALSE)

  # all pairwise contrasts (Bonferroni CI, Holm p)
  bfull <- c(0, b1); names(bfull) <- MODELS
  Vfull <- matrix(0, k, k, dimnames = list(MODELS, MODELS)); Vfull[-1, -1] <- sys_vcov(m1)
  zb <- qnorm(1 - 0.05 / (2 * length(pairs_list)))
  ctr <- t(sapply(pairs_list, function(pr) {
    L <- setNames(rep(0, k), MODELS); L[pr[2]] <- 1; L[pr[1]] <- -1
    est <- sum(L * bfull); se <- sqrt(drop(t(L) %*% Vfull %*% L))
    c(est = est, lo = est - zb * se, hi = est + zb * se, p = 2 * pnorm(-abs(est / se))) }))
  ctr <- cbind(ctr, p_holm = p.adjust(ctr[, "p"], "holm"))
  for (i in seq_along(pairs_list))
    say("  contrast %-38s OR %.2f (Bonferroni 95%% CI %.2f-%.2f), Holm p = %s", pair_lab[i],
        exp(ctr[i, "est"]), exp(ctr[i, "lo"]), exp(ctr[i, "hi"]), pfmt(ctr[i, "p_holm"]))
  clmm_res[[oc]] <- list(m1 = m1, lr = lr, vq = vq, po = po, b1 = b1, se1 = se1, ctr = ctr, bci = bci)
}
S3 <- do.call(rbind, S3_rows)

###############################################################################
# 6. Unpaired proportional-odds model (sensitivity; Table 6)
###############################################################################
polr_res <- list()
for (oc in names(outcomes)) {
  o <- outcomes[[oc]]; hdr(paste("Unpaired proportional-odds model:", oc))
```

```
  p1 <- clm(as.formula(paste(o$fac, "~ model")), data = d)
  p0 <- clm(as.formula(paste(o$fac, "~ 1")), data = d)
  lr <- 2 * (as.numeric(logLik(p1)) - as.numeric(logLik(p0)))
  r2 <- 1 - as.numeric(logLik(p1)) / as.numeric(logLik(p0))
  cf <- summary(p1)$coefficients[paste0("model", MODELS[-1]), ]
  say("LR chi2(4) = %.1f, p = %s, McFadden R2 = %.2f", lr, pfmt(pchisq(lr, 4, lower.tail = FALSE)), r2)
  for (j in 1:4) say("  %-16s OR %.2f (95%% CI %.2f-%.2f), p = %s", MODELS[j + 1], exp(cf[j, 1]),
                     exp(cf[j, 1] - 1.96 * cf[j, 2]), exp(cf[j, 1] + 1.96 * cf[j, 2]), pfmt(cf[j, 4]))
  polr_res[[oc]] <- list(or = exp(cf[, 1]), lr = lr, r2 = r2)
}

################################################################################
# 7. Ranking stability (question-level bootstrap)
################################################################################
hdr("Ranking stability, accuracy (bootstrap of questions)")
set.seed(SEED)
MA <- res$accuracy$M
first <- setNames(numeric(k), MODELS); top2 <- 0; last_cp <- 0
pair_order <- setNames(numeric(length(pairs_list)), pair_lab)
for (i in seq_len(N_BOOT_RANK)) {
  mm <- colMeans(MA[sample(nQ, replace = TRUE), , drop = FALSE])
  mx <- which(mm == max(mm)); first[mx] <- first[mx] + 1 / length(mx)
  o2 <- names(sort(mm, decreasing = TRUE))[1:2]
  top2 <- top2 + all(c("GPT-4", "GPT-4o") %in% o2)
  last_cp <- last_cp + (mm["Consensus Premium"] < min(mm[-1]))
  pair_order <- pair_order + sapply(pairs_list, function(pr) mm[pr[2]] > mm[pr[1]])
}
for (m in MODELS) say("  %-18s ranked first in %.1f%% of resamples", m, 100 * first[m] / N_BOOT_RANK)
say("  GPT-4o and GPT-4 in top two: %.1f%%; Consensus Premium last: %.1f%%",
    100 * top2 / N_BOOT_RANK, 100 * last_cp / N_BOOT_RANK)
for (i in seq_along(pair_lab)) say("  %-38s second ranked above first in %.1f%%", pair_lab[i], 100 *
pair_order[i] / N_BOOT_RANK)

################################################################################
# 8. Response length and rated quality (exploratory; Section 3.5)
################################################################################
hdr("Response length and rated quality (exploratory)")
d$log2w   <- log2(d$words)
d$log2w_c <- d$log2w - ave(d$log2w, d$question)
len_res <- list()
for (oc in names(outcomes)) {
  o <- outcomes[[oc]]
  a <- fit_clmm(as.formula(paste(o$fac, "~ log2w_c + domain + (1|question)")), d)
  b <- fit_clmm(as.formula(paste(o$fac, "~ log2w_c + domain + model + (1|question)")), d)
  se_of <- function(f) tryCatch(sqrt(vcov(f)["log2w_c", "log2w_c"]), error = function(e) NA)
  ea <- a$beta["log2w_c"]; sa <- se_of(a)
  eb <- b$beta["log2w_c"]; sb <- se_of(b)
  say("%s: OR per doubling of words %.1f (95%% CI %.1f-%.1f); + system %.1f (%.1f-%.1f)", oc,
      exp(ea), exp(ea - 1.96 * sa), exp(ea + 1.96 * sa), exp(eb), exp(eb - 1.96 * sb), exp(eb + 1.96 * sb))
  len_res[[oc]] <- c(exp(ea), exp(eb))
}

################################################################################
# 9. Table S4: paired pairwise comparisons
################################################################################
S4 <- data.frame(Comparison = pair_lab, check.names = FALSE)
for (oc in names(outcomes)) {
  ct <- clmm_res[[oc]]$ctr; r <- res[[oc]]; lab <- tools::toTitleCase(oc)
  S4[[paste0(lab, ": CLMM OR (Bonferroni 95% CI)")]] <-
    sprintf("%.2f (%.2f-%.2f)", exp(ct[, "est"]), exp(ct[, "lo"]), exp(ct[, "hi"]))
  S4[[paste0(lab, ": CLMM Holm p")]] <- pfmt(ct[, "p_holm"])
  S4[[paste0(lab, ": Conover Holm p")]] <- pfmt(r$con)
  S4[[paste0(lab, ": paired mean difference (95% CI)")]] <-
    sprintf("%.2f (%.2f to %.2f)", r$mdiff[, 1], r$mdiff[, 2], r$mdiff[, 3])
  S4[[paste0(lab, ": difference in % ", ifelse(oc == "accuracy", "good/excellent", "comprehensive/very"), "
(95% CI)")]] <-
    sprintf("%.1f (%.1f to %.1f)", 100 * r$pdiff[, 1], 100 * r$pdiff[, 2], 100 * r$pdiff[, 3])
  S4[[paste0(lab, ": exact McNemar Holm p")]] <- pfmt(r$mcn)
}
S4$`Words: Conover Holm p` <- pfmt(con_w)
S4$`Characters: Conover Holm p` <- pfmt(con_c)

################################################################################
# 10. Table S5 (guideline recommendations) and workbook export
################################################################################
s5_file <- file.path(DATA_DIR, "Table_S5_guideline_recommendations.csv")
S5 <- if (file.exists(s5_file)) read.csv(s5_file, stringsAsFactors = FALSE, check.names = FALSE, encoding =
"UTF-8") else NULL
if (!is.null(S5)) names(S5) <- c("Question", "Domain", "Recommendation no.", "Question (English)",
                                 "Recommendation (German original)", "Recommendation (English translation)",
                                 "Grade of recommendation", "Level of evidence", "Consensus
strength")[seq_along(S5)]

titles <- list(
```

```
  S1 = c("Table S1. Word and character counts for every clinical question, every model and the corresponding
DGOU guideline text.",
         "Each question was mapped to one numbered recommendation of the EKIT-Hip guideline (AWMF 187-001).
Words and characters were counted programmatically. SD, standard deviation."),
  S2 = c("Table S2. Response length by guideline domain.",
         "Values are mean ± SD across the questions of each domain. Ratio, model length divided by guideline
text length per question. Friedman test across the five systems within each domain."),
  S3 = c("Table S3. Cumulative-link mixed-model estimates, including sensitivity models with domain.",
         "Odds ratios for a higher rating category relative to Consensus Premium; random intercept for
question. Cluster-bootstrap CIs: 2000 resamples of the 26 questions. Sensitivity models add domain as a fixed
effect or as a random intercept. PO, proportional-odds (likelihood-ratio test against a nominal-effects
model)."),
  S4 = c("Table S4. Paired pairwise comparisons (mixed-model contrasts and Conover post hoc tests).",
         "OR > 1 and positive differences favour the second-named system. CLMM contrasts with Bonferroni-
adjusted 95% CIs and Holm-adjusted Wald p-values; Conover post hoc tests after the Friedman test (Holm);
paired differences with question-level cluster-bootstrap 95% CIs; exact McNemar tests (Holm)."),
  S5 = c("Table S5. Guideline recommendations with grade of recommendation, level of evidence, consensus
strength, English translation and corresponding question.",
         "Source: S3 guideline 'Evidenz- und konsensbasierte Indikationskriterien zur Hüfttotalendoprothese
bei Coxarthrose (EKIT-Hüfte)', AWMF register no. 187-001, version 1.0 (2021). English translations by the
authors."))

wb <- createWorkbook()
hs <- createStyle(textDecoration = "bold", border = "bottom", wrapText = TRUE, valign = "top")
add_tab <- function(name, df, tt) {
  addWorksheet(wb, name)
  writeData(wb, name, tt[1], startRow = 1); addStyle(wb, name, createStyle(textDecoration = "bold"), 1, 1)
  writeData(wb, name, df, startRow = 3, headerStyle = hs)
  writeData(wb, name, tt[2], startRow = nrow(df) + 5)
  setColWidths(wb, name, 1:ncol(df), widths = pmax(12, pmin(45, nchar(names(df)) * 0.9)))
  write.csv(df, file.path(OUT_DIR, paste0("Table_", name, ".csv")), row.names = FALSE, fileEncoding = "UTF-8")
}
add_tab("S1", S1_out, titles$S1); add_tab("S2", S2, titles$S2)
add_tab("S3", S3, titles$S3);     add_tab("S4", S4, titles$S4)
if (!is.null(S5)) add_tab("S5", S5, titles$S5)
saveWorkbook(wb, file.path(OUT_DIR, "Supplementary_Tables_S1-S5.xlsx"), overwrite = TRUE)

###############################################################################
# 11. Check every recomputed value against the manuscript
###############################################################################
chk <- list()
ck <- function(item, got, expected, tol) chk[[length(chk) + 1]] <<- data.frame(
  item = item, manuscript = expected, recomputed = round(as.numeric(got), 4),
  status = ifelse(abs(as.numeric(got) - expected) <= tol + 1e-9, "PASS", "FAIL"))

# Table 2
exp_len <- list(
  "Consensus Premium" = c(32.4, 11.5, 34.5, 16, 58, 217.2, 80.4, 224.5, 1.29, 0.60),
  "Gemini Advanced"   = c(40.0, 13.7, 41.5, 19, 66, 271.5, 99.4, 270.0, 1.59, 0.80),
  "GPT-4"             = c(62.1, 24.3, 64.5, 25, 133, 400.3, 141.4, 394.5, 2.53, 1.41),
  "GPT-4o mini"       = c(57.5, 17.2, 58.0, 25, 78, 384.5, 115.9, 397.0, 2.28, 0.93),
  "GPT-4o"            = c(77.8, 24.9, 78.0, 35, 118, 512.1, 147.3, 520.5, 3.19, 1.57))
for (m in MODELS) { e <- exp_len[[m]]
  got <- c(mean(W[, m]), sd(W[, m]), median(W[, m]), min(W[, m]), max(W[, m]),
           mean(C[, m]), sd(C[, m]), median(C[, m]), mean(RT[, m]), sd(RT[, m]))
  lb <- c("words mean", "words SD", "words median", "words min", "words max",
          "chars mean", "chars SD", "chars median", "ratio mean", "ratio SD")
  tl <- c(.05, .05, .05, 0, 0, .05, .05, .05, .005, .005)
  for (i in seq_along(e)) ck(paste("Table 2", m, lb[i]), got[i], e[i], tl[i]) }
ck("Table 2 guideline words mean", mean(glw), 27.8, .05); ck("Table 2 guideline words SD", sd(glw), 10.0, .05)
ck("Table 2 guideline words median", median(glw), 25.0, .05)
ck("Table 2 guideline words min", min(glw), 14, 0); ck("Table 2 guideline words max", max(glw), 56, 0)
ck("Table 2 guideline chars mean", mean(glc), 165.3, .05); ck("Table 2 guideline chars SD", sd(glc),
69.1, .05)
ck("Table 2 guideline chars median", median(glc), 146.5, .05)
ck("Wilcoxon Consensus Premium vs guideline p", wil["Consensus Premium"], 0.141, .0005)
ck("Wilcoxon Gemini Advanced vs guideline p", wil["Gemini Advanced"], 0.0009, .00005)
ck("Friedman words chi2", fw$statistic, 47.3, .05); ck("Friedman chars chi2", fc$statistic, 46.4, .05)
d5 <- levels(droplevels(d$question[d$domain == "D5"]))
ck("D5 GPT-4o words mean", mean(W[d5, "GPT-4o"]), 94.8, .05); ck("D5 GPT-4o words SD", sd(W[d5, "GPT-4o"]),
20.7, .05)
ck("D5 guideline words mean", mean(glw[d5]), 20.1, .05); ck("D5 guideline words SD", sd(glw[d5]), 3.9, .05)

# Tables 3 and 4
exp_cnt <- list(accuracy = list("Consensus Premium" = c(2, 9, 14, 1), "Gemini Advanced" = c(1, 5, 11, 9),
                                "GPT-4" = c(0, 1, 8, 17), "GPT-4o mini" = c(0, 5, 9, 12), "GPT-4o" = c(0, 1,
6, 19)),
                comprehensiveness = list("Consensus Premium" = c(2, 3, 13, 8, 0), "Gemini Advanced" = c(1, 3,
9, 11, 2),
                                         "GPT-4" = c(0, 1, 3, 15, 7), "GPT-4o mini" = c(0, 2, 5, 13, 6), "GPT-
4o" = c(0, 1, 2, 11, 12)))
exp_ms <- list(accuracy = rbind(c(2.54, .71), c(3.08, .84), c(3.62, .57), c(3.27, .78), c(3.69, .55)),
               comprehensiveness = rbind(c(3.04, .87), c(3.38, .94), c(4.08, .74), c(3.88, .86),
c(4.31, .79)))
```

```
exp_mr <- list(accuracy = c(35.0, 58.4, 81.8, 66.3, 85.9), comprehensiveness = c(38.8, 51.8, 78.2, 70.6,
88.1))
for (oc in names(outcomes)) { M <- res[[oc]]$M
  for (j in seq_along(MODELS)) { m <- MODELS[j]
    cnt <- tabulate(M[, m], outcomes[[oc]]$K)
    for (c_ in seq_along(cnt)) ck(sprintf("%s %s count of category %d", oc, m, c_), cnt[c_],
exp_cnt[[oc]][[m]][c_], 0)
    ck(sprintf("%s %s mean", oc, m), mean(M[, m]), exp_ms[[oc]][j, 1], .005)
    ck(sprintf("%s %s SD", oc, m), sd(M[, m]), exp_ms[[oc]][j, 2], .005)
    ck(sprintf("%s %s mean rank", oc, m), res[[oc]]$mean_rank[m], exp_mr[[oc]][j], .05) } }
ck("Accuracy Friedman chi2", res$accuracy$fr$statistic, 41.7, .05)
ck("Comprehensiveness Friedman chi2", res$comprehensiveness$fr$statistic, 35.6, .05)
ck("Accuracy Kruskal-Wallis H", res$accuracy$kw$statistic, 35.6, .05)
ck("Comprehensiveness Kruskal-Wallis H", res$comprehensiveness$kw$statistic, 33.0, .05)
ck("Accuracy epsilon^2", res$accuracy$eps2, .25, .005); ck("Comprehensiveness epsilon^2",
res$comprehensiveness$eps2, .23, .005)
ck("Cochran's Q good/excellent", res$accuracy$cq["Q"], 22.2, .05)
ck("Cochran's Q highly comprehensive", res$comprehensiveness$cq["Q"], 30.0, .05)
ck("Cochran's Q major errors", cqm["Q"], 5.33, .005)
ck("Unpaired chi2 good/excellent", res$accuracy$ct$statistic, 17.6, .05)
ck("Unpaired chi2 highly comprehensive", res$comprehensiveness$ct$statistic, 27.5, .05)
ck("Accuracy TOST paired difference GPT-4o - GPT-4", res$accuracy$tost["diff"], .08, .005)
ck("Comprehensiveness paired difference GPT-4o - GPT-4", res$comprehensiveness$tost["diff"], .23, .005)
# Table 5 Cliff's delta
exp_cd <- list(accuracy = c(.36, .73, .48, .77, .36, .12, .42, -.24, .07, .30),
               comprehensiveness = c(.21, .63, .50, .72, .42, .30, .55, -.12, .19, .29))
for (oc in names(outcomes)) for (i in seq_along(pair_lab))
  ck(sprintf("%s Cliff's delta %s", oc, pair_lab[i]), res[[oc]]$cd[i], exp_cd[[oc]][i], .005)
# CLMM
ck("Accuracy CLMM LR chi2", clmm_res$accuracy$lr$LR.stat[2], 49.0, .05)
ck("Comprehensiveness CLMM LR chi2", clmm_res$comprehensiveness$lr$LR.stat[2], 44.3, .05)
ck("Accuracy CLMM question variance", clmm_res$accuracy$vq, 1.73, .005)
ck("Comprehensiveness CLMM question variance", clmm_res$comprehensiveness$vq, 1.37, .005)
ck("Accuracy PO test chi2", clmm_res$accuracy$po["stat"], 6.12, .005)
ck("Comprehensiveness PO test chi2", clmm_res$comprehensiveness$po["stat"], 5.95, .005)
exp_or <- list(accuracy = c(5.37, 35.36, 9.45, 51.80), comprehensiveness = c(2.51, 15.41, 8.77, 33.92))
for (oc in names(outcomes)) for (j in 1:4)
  ck(sprintf("%s CLMM OR %s", oc, MODELS[j + 1]), exp(clmm_res[[oc]]$b1[j]), exp_or[[oc]][j], .005)
# unpaired proportional-odds (Table 6)
exp_po <- list(accuracy = c(3.62, 15.22, 5.81, 21.33), comprehensiveness = c(1.97, 9.58, 6.10, 19.41))
for (oc in names(outcomes)) { for (j in 1:4)
  ck(sprintf("%s unpaired OR %s", oc, MODELS[j + 1]), polr_res[[oc]]$or[j], exp_po[[oc]][j], .005) }
ck("Accuracy unpaired LR chi2", polr_res$accuracy$lr, 37.9, .05)
ck("Comprehensiveness unpaired LR chi2", polr_res$comprehensiveness$lr, 36.7, .05)
ck("Accuracy McFadden R2", polr_res$accuracy$r2, .13, .005)
ck("Comprehensiveness McFadden R2", polr_res$comprehensiveness$r2, .11, .005)
# length and quality
ck("Accuracy OR per doubling of words", len_res$accuracy[1], 16.2, .05)
ck("Comprehensiveness OR per doubling of words", len_res$comprehensiveness[1], 33.5, .05)
ck("Accuracy OR per doubling, + system", len_res$accuracy[2], 10.3, .05)
ck("Comprehensiveness OR per doubling, + system", len_res$comprehensiveness[2], 26.9, .05)

chk <- do.call(rbind, chk)
write.csv(chk, file.path(OUT_DIR, "manuscript_check.csv"), row.names = FALSE)
hdr("Check against manuscript")
say("%d of %d reported values reproduced (%d FAIL). See output/manuscript_check.csv.",
    sum(chk$status == "PASS"), nrow(chk), sum(chk$status == "FAIL"))
if (any(chk$status == "FAIL")) print(chk[chk$status == "FAIL", ], row.names = FALSE)

hdr("Session info"); capture.output(sessionInfo()) |> paste(collapse = "\n") |> say()
close(log_con)
```
