# Supplementary material for: Guideline-Concordant Answer Quality of Five Large Language Models on Indication Criteria for Total Hip Arthroplasty in Hip Osteoarthritis: A Single-Run Benchmark Against the 2021 German S3 Guideline (EKIT-Hip)
Source: J Clin Med. 2026 Oct 14;15(20):7944. doi: 10.3390/jcm15207944 (PMC13706208; doi:10.3390/jcm15207944)
Supplement: Supplementary file 1 [file jcm-15-07944-s001.zip › jcm-4566380-supplementary.pdf]

Supplementary materials

Table S1. Word and character counts for every clinical question, every model and the corresponding DGOU guideline text.

Question	DGOU section	DGOU (words)	Consensus Premium (words)	Gemini Advanced (words)	GPT-4 (words)	GPT-4o mini (words)	GPT-4o (words)	DGOU (characters)	Consensus Premium (characters)	Gemini Advanced (characters)	GPT-4 (characters)	GPT-4o mini (characters)	GPT-4o (characters)
Q1	4.1.1.	31	28	20	67	25	68	186	173	145	409	178	428
Q2	4.1.2.	27	58	47	44	64	37	174	392	372	328	428	278
Q3	4.1.3.	40	24	34	41	45	61	211	163	226	248	290	410
Q4	4.1.4.	28	24	34	73	76	82	139	138	212	384	452	469
Q5	4.1.5	22	37	35	133	71	108	150	218	258	792	454	654
Q6	4.1.6.	23	40	24	46	78	103	137	285	179	307	540	693
Q7	4.1.7.	21	27	55	71	76	92	110	174	311	459	512	622
Q8	4.1.8.	22	16	34	25	38	35	101	119	265	212	293	260
Q9	4.2.1	36	20	49	36	49	35	216	121	319	272	317	251
Q10	4.2.2.	21	44	66	34	34	41	149	314	506	232	205	298
Q11	4.2.3.	43	18	62	41	78	71	257	130	431	263	555	501
Q12	4.2.4.	31	53	30	49	59	74	178	326	226	339	383	464
Q13	4.3.1.	26	34	28	58	72	68	198	289	233	402	497	507
Q14	4.3.2.	49	36	45	79	37	58	342	282	312	504	270	410
Q15	4.3.3.	29	19	44	45	57	74	193	139	317	326	411	508
Q16	4.4.1. (2)	24	27	43	31	51	82	122	171	275	202	355	543
Q17	4.4.3.	56	43	54	89	78	72	360	327	383	627	580	533
Q18	4.4.4.	32	44	47	65	55	105	164	253	325	390	373	661
Q19	4.5.1.	25	35	40	49	37	118	134	242	294	347	289	763
Q20	4.5.2.	18	28	63	64	28	52	101	187	410	399	163	331
Q21	4.5.3.	17	16	25	70	45	91	111	85	138	405	267	561
Q22	4.5.4.	21	38	19	70	68	109	105	252	106	435	424	668
Q23	4.5.5.	14	37	49	70	56	84	82	236	291	471	376	553
Q24	4.5.6.	19	44	44	97	65	111	110	281	242	623	443	723
Q25	4.5.7.	22	17	20	93	74	92	125	118	134	586	458	601

Question	DGOU section	DGOU (words)	Consensus Premium (words)	Gemini Advanced (words)	GPT-4 (words)	GPT-4o mini (words)	GPT-4o (words)	DGOU (characters)	Consensus Premium (characters)	Gemini Advanced (characters)	GPT-4 (characters)	GPT-4o mini (characters)	GPT-4o (characters)
Q26	4.5.8.	25	36	28	75	78	101	144	231	150	447	484	624

DGOU, German Society for Orthopaedics and Trauma Surgery.

Table S2. Response length by guideline domain.

Domain	n questions	DGOU words	Consensus Premium words, mean ± SD	Gemini Advanced words, mean ± SD	GPT-4 words, mean ± SD	GPT-4o mini words, mean ± SD	GPT-4o words, mean ± SD	Friedman p
D1 — Diagnosis and imaging	8	26.8	31.8 ± 13.0	35.4 ± 11.3	62.5 ± 33.1	59.1 ± 20.4	73.2 ± 28.0	0.004
D2 — Assessment of the patient's level of suffering	4	32.8	33.8 ± 17.4	51.8 ± 16.2	40.0 ± 6.7	55.0 ± 18.5	55.2 ± 20.1	0.442
D3 — Evaluation of alternative therapeutic options	3	34.7	29.7 ± 9.3	39.0 ± 9.5	60.7 ± 17.2	55.3 ± 17.6	66.7 ± 8.1	0.102
D4 — Contraindications	3	37.3	38.0 ± 9.5	48.0 ± 5.6	61.7 ± 29.1	61.3 ± 14.6	86.3 ± 16.9	0.074
D5 — Optimisation of modifiable risk factors	8	20.1	31.4 ± 10.2	36.0 ± 15.6	73.5 ± 15.4	56.4 ± 18.1	94.8 ± 20.7	<0.0001

Values are mean ± standard deviation words per response. Global comparison within each domain by Friedman test.

Table S3. Cumulative-link mixed models (random intercept for question) for accuracy and comprehensiveness: odds ratios for a higher rating relative to Consensus Premium, with Wald and question-level cluster-bootstrap (2000 resamples) 95% confidence intervals, and sensitivity models including guideline domain.

Outcome	System (vs Consensus Premium)	OR	Wald 95% CI	Cluster-bootstrap 95% CI	p	OR, domain as fixed effect	OR, domain as random effect
Accuracy	Gemini Advanced	5.37	1.70–16.91	1.76–19.83	0.004	5.44	5.33
Accuracy	GPT-4	35.36	9.17–136.39	16.53–137.30	<0.0001	34.90	35.09
Accuracy	GPT-4o mini	9.45	2.87–31.12	3.16–47.24	0.0002	9.36	9.34
Accuracy	GPT-4o	51.80	12.43–215.88	17.85–338.80	<0.0001	51.13	51.40
Comprehensiveness	Gemini Advanced	2.51	0.87–7.24	0.91–8.18	0.089	2.55	2.53
Comprehensiveness	GPT-4	15.41	4.77–49.72	6.94–56.11	<0.0001	15.63	15.40
Comprehensiveness	GPT-4o mini	8.77	2.79–27.57	2.73–45.02	0.0002	8.73	8.73
Comprehensiveness	GPT-4o	33.92	9.68–118.86	11.61–166.51	<0.0001	33.83	33.80

Accuracy: likelihood-ratio $\chi^2(4) = 49.0$, $p < 0.0001$; SD of question intercept 1.31; proportional-odds test $\chi^2(8) = 6.12$, $p = 0.63$; Friedman $\chi^2(4) = 41.7$, $p < 0.0001$, Kendall's $W = 0.40$; Cochran's Q (good or excellent) = 22.2, $p = 0.0002$; Cochran's Q (major error) = 5.33, $p = 0.25$. Comprehensiveness: likelihood-ratio $\chi^2(4) = 44.3$, $p < 0.0001$; SD of question intercept 1.17; proportional-odds test $\chi^2(12) = 5.95$, $p = 0.92$; Friedman $\chi^2(4) = 35.6$, $p < 0.0001$, Kendall's $W = 0.34$; Cochran's Q (comprehensive or very comprehensive) = 30.0, $p < 0.0001$. CI, confidence interval; OR, odds ratio.

Table S4. Paired pairwise comparisons: mixed-model contrasts (odds ratio of the first-named relative to the second-named system, Bonferroni-adjusted 95% CI, Holm-adjusted p) and Conover post-hoc tests after the Friedman test (Holm-adjusted p).

Comparison	Accuracy: OR (Bonferroni 95% CI)	Accuracy: Holm p (mixed model)	Accuracy: Holm p (Conover)	Comprehensiveness: OR (Bonferroni 95% CI)	Comprehensiveness: Holm p (mixed model)	Comprehensiveness: Holm p (Conover)
Gemini Advanced vs Consensus Premium	5.37 (1.04–27.76)	0.021	0.008	2.51 (0.55–11.46)	0.268	0.221
GPT-4 vs Consensus Premium	35.36 (5.11–244.43)	<0.0001	<0.0001	15.41 (2.88–82.49)	<0.0001	<0.0001
GPT-4o mini vs Consensus Premium	9.45 (1.71–52.08)	0.002	0.0003	8.77 (1.70–45.22)	0.001	0.001
GPT-4o vs Consensus Premium	51.80 (6.71–400.04)	<0.0001	<0.0001	33.92 (5.63–204.35)	<0.0001	<0.0001
GPT-4 vs Gemini Advanced	6.59 (1.15–37.77)	0.015	0.003	6.14 (1.25–30.07)	0.008	0.006
GPT-4o mini vs Gemini Advanced	1.76 (0.35–8.76)	0.644	0.543	3.50 (0.72–16.93)	0.104	0.173
GPT-4o vs Gemini Advanced	9.65 (1.52–61.26)	0.004	0.002	13.52 (2.48–73.63)	0.0001	0.0001
GPT-4o mini vs GPT-4	0.27 (0.05–1.49)	0.094	0.045	0.57 (0.12–2.67)	0.309	0.376
GPT-4o vs GPT-4	1.47 (0.23–9.21)	0.644	0.846	2.20 (0.46–10.44)	0.309	0.376
GPT-4o vs GPT-4o mini	5.48 (0.89–33.70)	0.034	0.036	3.87 (0.77–19.51)	0.095	0.076

An odds ratio above 1 indicates higher odds of a higher rating for the first-named system.

Table S5. Recommendations of the 2021 S3 guideline “Evidenz- und konsensbasierte Indikationskriterien zur Hüfttotalendoprothese bei Coxarthrose (EKIT-Hüfte)” (AWMF register no. 187-001), with grade of recommendation, level of evidence, consensus strength, English translation and the corresponding question of the present study.

Rec. no.	Thematic complex	Grade	Level of evidence	Consensus strength	Recommendation (German original)	English translation	Question
1.1	1 Diagnosis	EK	Konsensbasiert	Starker Konsens (96%)	Die Diagnose Coxarthrose soll klinisch anhand einer spezifischen Anamnese (Hüftschmerzen, Morgensteifigkeit < 60min) und einer spezifischen klinischen Untersuchung (schmerzhafte Innenrotation und eingeschränkte Flexion) gesichert werden.	The diagnosis of hip osteoarthritis shall be established clinically on the basis of a specific history (hip pain, morning stiffness < 60 min) and a specific clinical examination (painful internal rotation and restricted flexion).	Q1
1.2	1 Diagnosis	A	2+	Starker Konsens (96%)	Vor Indikationsstellung zur Hüft-TEP sollen relevante Differentialdiagnosen der Coxarthrose ausgeschlossen werden. Dies gilt insbesondere für jüngere Patient*innen, bei denen evtl. noch gelenkerhaltende Operationen möglich sind.	Before the indication for THA is established, relevant differential diagnoses of hip osteoarthritis shall be excluded. This applies in particular to younger patients, in whom joint-preserving surgery may still be possible.	Q2
1.3	1 Diagnosis	EK	Konsensbasiert	Starker Konsens (100%)	Zur Diagnosesicherung und Beurteilung des Ausmaßes der degenerativen Veränderungen soll vor Indikationsstellung zu einer Hüft-TEP eine Bildgebung mittels konventionellem Röntgen (Beckenübersicht und eine zweite Ebene des betroffenen Hüftgelenks) vorliegen.	To confirm the diagnosis and assess the extent of degenerative changes, conventional radiographs (anteroposterior pelvis and a second plane of the affected hip) shall be available before the indication for THA is established.	Q3

Rec. no.	Thematic complex	Grade	Level of evidence	Consensus strength	Recommendation (German original)	English translation	Question
1.4	1 Diagnosis	EK	4	Starker Konsens (100%)	Eine weiterführende Bildgebung mittels MRT und / oder CT im Rahmen der Indikationsstellung zur Hüft-TEP soll nur bei Diskrepanz zwischen klinischem und röntgenologischem Befund erfolgen.	Further imaging with MRI and/or CT as part of establishing the indication for THA shall be performed only if clinical and radiographic findings are discrepant.	Q4
1.5	1 Diagnosis	EK	Konsensbasiert	Konsens (93%)	Bei zweifelhafter oder unklarer Diagnosestellung kann vor Empfehlung zur endoprothetischen Versorgung eine bildgestützte intraartikuläre Infiltration mit einem Lokalanästhetikum erfolgen.	If the diagnosis is doubtful or unclear, an image-guided intra-articular injection of a local anaesthetic may be performed before arthroplasty is recommended.	Q5
1.6	1 Diagnosis	EK	Konsensbasiert	Starker Konsens (100%)	In der operierenden Einrichtung soll durch eine spezifische Anamnese, klinische Untersuchung und Prüfung der Röntgenaufnahmen der Schweregrad der Coxarthrose beurteilt werden.	In the operating institution, the severity of hip osteoarthritis shall be assessed by a specific history, clinical examination and review of the radiographs.	Q6
1.7	1 Diagnosis	A	2+	Starker Konsens (96%)	Eine Hüft-TEP-Operation soll nur bei radiologisch nachgewiesener fortgeschrittener Coxarthrose (Kellgren & Lawrence Grad 3 oder 4) erfolgen.	THA shall be performed only in radiologically confirmed advanced hip osteoarthritis (Kellgren & Lawrence grade 3 or 4).	Q7
1.8	1 Diagnosis	B	2++	Konsens (93%)	Bei Nachweis einer Hüftkopfnekrose sollte ab ARCO Stadium III c die Indikation zur Implantation einer Hüft-TEP überprüft werden.	If osteonecrosis of the femoral head is present, the indication for THA should be reviewed from ARCO stage IIIc onwards.	Q8
2.1	2 Burden of disease	A	1b	Starker Konsens (100%)	Vor Indikationsstellung zu einer Hüft-TEP soll der Leidensdruck der Patient*innen durch die Erhebung folgender Coxarthrose-bedingter Symptome beurteilt werden: Schmerzen, Einschränkungen der Funktion und der Aktivitäten des täglichen Lebens, Einschränkungen der gesundheitsbezogenen Lebensqualität.	Before the indication for THA is established, the patient's burden of disease shall be assessed by recording the following osteoarthritis-related symptoms: pain, limitations of function and activities of daily living, and limitations of health-related quality of life.	Q9
2.2	2 Burden of disease	A	2+	Starker Konsens (100%)	Zur Beurteilung des Leidensdruckes der Patient*innen sollen validierte Instrumente patientenberichteter Outcomes genutzt werden. Dazu bieten sich krankheitsspezifische und generische Instrumente an.	Validated patient-reported outcome instruments shall be used to assess the patient's burden of disease; both disease-specific and generic instruments are suitable.	Q10
2.3	2 Burden of disease	A	3	Starker Konsens (100%)	Die Indikationsstellung zur Hüft-TEP soll in Betracht gezogen werden, wenn Patient*innen, trotz vorangegangener konservativer Therapie, über hohen subjektiven Leidensdruck hinsichtlich hüftbezogener Beschwerden (Schmerzen, Funktionseinschränkungen, Einschränkungen bei den Aktivitäten des täglichen Lebens) und der gesundheitsbezogenen Lebensqualität berichten.	THA shall be considered if, despite previous conservative therapy, patients report a high subjective burden of hip-related symptoms (pain, functional limitations, limitations in activities of daily living) and of impaired health-related quality of life.	Q11
2.4	2 Burden of disease	A	2+	Starker Konsens (100%)	Im Aufklärungsgespräch sollen unter Berücksichtigung der individuellen Symptomausprägung die Vor- und Nachteile einer frühen bzw. späten Indikationsstellung zur Hüft-TEP mit den Patient*innen abgewogen werden.	In the informed-consent discussion, the advantages and disadvantages of an early versus a late indication for THA shall be weighed with the patient, taking the individual severity of symptoms into account.	Q12
3.1	3 Alternative treatment	A	1+	Starker Konsens (96%)	Vor der Indikationsstellung zur Hüft-TEP sollen Patient*innen mit symptomatischer Coxarthrose mit einer Kombination aus medikamentöser und nicht-medikamentöser konservativer Therapie behandelt werden.	Before the indication for THA is established, patients with symptomatic hip osteoarthritis shall be treated with a combination of pharmacological and non-pharmacological conservative therapy.	Q13

Rec. no.	Thematic complex	Grade	Level of evidence	Consensus strength	Recommendation (German original)	English translation	Question
3.2	3 Alternative treatment	A	1+	Konsens (89%)	Vor der Indikationsstellung zur Hüft-TEP-Operation sollen Patient*innen mindestens die Kernelemente der nicht-medikamentösen konservativen Therapie durchgeführt haben bzw. sollen empfohlen werden: Patient*innenedukation, Bewegungstherapie und Förderung der körperlichen Aktivität, Gewichtsreduktion bei Patient*innen mit Übergewicht und Adipositas.	Before the indication for THA is established, patients shall have undergone, or shall be recommended, at least the core elements of non-pharmacological conservative therapy: patient education, exercise therapy and promotion of physical activity, and weight reduction in patients with overweight or obesity.	Q14
3.3	3 Alternative treatment	B	1+	Konsens (84%)	Wenn trotz leitliniengerechter konservativer Therapiemaßnahmen über mindestens drei Monate die Patient*innen über einen hohen subjektiven Leidensdruck berichten, sollte die Indikationsstellung zur Hüft-TEP erfolgen.	If patients report a high subjective burden of disease despite guideline-based conservative treatment for at least three months, the indication for THA should be established.	Q15
4.1	4 Contraindications	A	2+	Konsens (93%)	Vor einer Hüft-TEP-Operation soll eine vorangegangene Infektion des zu operierenden Hüftgelenkes auf noch bestehende Aktivität geprüft werden.	Before THA, a previous infection of the hip to be operated on shall be checked for persisting activity.	Q16
4.2	4 Contraindications	A	2-	Konsens (85%)	Vor einer Hüft-TEP-Operation soll eine aktive Infektion (insbesondere von Gelenken, Weichteilen oder hämatogen streuend) ausgeheilt sein.	Before THA, any active infection (in particular of joints or soft tissues, or with haematogenous spread) shall have resolved.	—
4.3	4 Contraindications	EK	Konsensbasiert	Konsens (93%)	Bei akuten oder chronischen Begleiterkrankungen, die mit einem erhöhten Sterblichkeitsrisiko verbunden sind, soll durch Operateur*innen die Empfehlung zur Durchführung bzw. dem Zeitpunkt der Hüft-TEP-Operation nach anästhesiologischer und gegebenenfalls fachinternistischer Risikoeinschätzung getroffen werden.	In patients with acute or chronic comorbidities associated with an increased risk of death, the surgeon shall decide whether and when to perform THA after anaesthesiological and, where appropriate, specialist internal-medicine risk assessment.	Q17
4.4	4 Contraindications	A	2++/2-	Konsens (92%)	Bei einem BMI ≥ 40 kg/m² soll aufgrund der deutlich erhöhten Komplikationsgefahr eine besonders kritische Abwägung von Nutzen und Risiken der Hüft-TEP-Operation erfolgen.	In patients with a BMI ≥ 40 kg/m², the benefits and risks of THA shall be weighed particularly critically because of the markedly increased risk of complications.	Q18
5.1	5 Modifiable risk factors	EK	2+	Starker Konsens (100%)	Bei Vorliegen modifizierbarer Risikofaktoren sollen Patient*innen über die Möglichkeit aufgeklärt werden, die Hüft-TEP-Operation zu verschieben, um diese Risiken zu minimieren.	If modifiable risk factors are present, patients shall be informed that THA can be postponed in order to minimise these risks.	Q19
5.1.1	5 Modifiable risk factors	A	2++	Starker Konsens (100%)	Raucher*innen soll empfohlen werden, den Nikotinkonsum mindestens einen Monat vor geplanter Hüft-TEP-Operation zu beenden.	Smokers shall be advised to stop nicotine consumption at least one month before planned THA.	Q20
5.1.2a	5 Modifiable risk factors	A	2++	Starker Konsens (96%)	Bei Patient*innen mit Diabetes mellitus sollen die Blutzuckerwerte vor einer Hüft-TEP-Operation bestmöglich eingestellt sein.	In patients with diabetes mellitus, blood glucose shall be optimally controlled before THA.	Q21
5.1.2b	5 Modifiable risk factors	EK	2++	Starker Konsens (96%)	Ein HbA1c-Wert unter 8% sollte angestrebt werden.	An HbA1c below 8% should be targeted.	Q21
5.1.3	5 Modifiable risk factors	B	2++/2+	Konsens (92%)	Bei einem BMI ≥ 30 kg/m2 sollte Patient*innen empfohlen werden, ihr Gewicht vor der Hüft-TEP-Operation zu reduzieren.	Patients with a BMI ≥ 30 kg/m² should be advised to reduce their weight before THA.	Q22
5.1.4	5 Modifiable risk factors	A	2+	Konsens (89%)	Eine asymptomatische Bakteriurie soll nicht wegen einer geplanten Hüft-TEP-Operation behandelt werden.	Asymptomatic bacteriuria shall not be treated because of planned THA.	Q23

Rec. no.	Thematic complex	Grade	Level of evidence	Consensus strength	Recommendation (German original)	English translation	Question
5.1.5	5 Modifiable risk factors	EK	2+	Konsens (89%)	Bei Verdacht auf eine psychische Erkrankung sollte den Patient*innen vor einer Hüft-TEP-Operation eine fachspezifische Abklärung empfohlen werden.	If a mental disorder is suspected, patients should be advised to undergo specialist assessment before THA.	Q24
5.1.6	5 Modifiable risk factors	A	1-/2+	Konsens (90%)	Vor einer Hüft-TEP-Operation soll eine Anämiediagnostik erfolgen und im Falle einer behandlungsbedürftigen Anämie eine Optimierung vorgenommen werden.	Before THA, anaemia shall be investigated and, if anaemia requiring treatment is present, it shall be corrected.	Q25
5.1.7	5 Modifiable risk factors	B	2++	Konsens (88%)	Nach einer intraartikulären Injektion von Cortikosteroiden sollte eine Hüft-TEP-Operation frühestens nach 6 Wochen erfolgen, zu empfehlen jedoch erst nach 3 Monaten.	After an intra-articular corticosteroid injection, THA should not be performed earlier than 6 weeks and preferably not before 3 months.	Q26
6.1	6 Shared decision-making	EK	Orientieren de Recherche	Konsens (93%)	Patient*innen sollen dazu angeregt werden, individuelle Therapieziele zu formulieren. Die gemeinsam erarbeiteten Therapieziele sollten dokumentiert werden.	Patients shall be encouraged to formulate individual treatment goals. The jointly developed goals should be documented.	—
6.2	6 Shared decision-making	EK	Orientieren de Recherche	Starker Konsens (100%)	Patient*innen sollen darüber aufgeklärt werden, inwiefern sich die individuellen Therapieziele durch eine Hüft-TEP oder alternative Therapieoptionen realisieren lassen.	Patients shall be informed to what extent their individual treatment goals can be achieved by THA or by alternative treatment options.	—
6.3	6 Shared decision-making	EK	Orientieren de Recherche	Starker Konsens (100%)	Für das Beratungs- und Aufklärungsgespräch sollen patientenverständliche Informationsmaterialien genutzt werden.	Patient-friendly information materials shall be used for counselling and informed-consent discussions.	—
6.4	6 Shared decision-making	EK	Orientieren de Recherche	Starker Konsens (100%)	Für die gemeinsam getroffene Entscheidung zur Hüft-TEP sollen sich Patient*innen und Operateur*innen darin einig sein, dass der zu erwartende Nutzen der Therapie die möglichen Risiken überwiegt.	For a shared decision on THA, patients and surgeons shall agree that the expected benefit of treatment outweighs the possible risks.	—

Recommendation texts, grades, levels of evidence and consensus strengths are reproduced from the guideline (reference 32). Grade A, strong recommendation (“soll”); grade B, recommendation (“sollte”); EK, expert consensus. Questions follow the order of the recommendations in the guideline; the section numbers listed for each question in Table S1 refer to this sequence. Recommendation 4.2 and the recommendations of thematic complex 6 (shared decision-making) were not queried. THA, total hip arthroplasty.

Data S1. Question-level consensus ratings (accuracy and comprehensiveness) and response lengths for all 130 responses, with guideline domain (file: Data_S1_question_level_ratings.csv).

Code S1. R code for the cumulative-link mixed models, paired sensitivity analyses (Friedman with Conover, Cochran’s Q, McNemar), cluster bootstrap, equivalence analysis and exploratory length analysis (file: Code_S1_analysis.R).
